# The proliferation role of LH on porcine primordial germ cell‐like cells (pPGCLCs) through ceRNA network construction

**DOI:** 10.1002/ctm2.560

**Published:** 2021-10-14

**Authors:** Ming‐Yu Zhang, Yu Tian, Shu‐Er Zhang, Hong‐Chen Yan, Wei Ge, Bao‐Quan Han, Zi‐Hui Yan, Shun‐Feng Cheng, Wei Shen

**Affiliations:** ^1^ College of Life Sciences, Key Laboratory of Animal Reproduction and Biotechnology in Universities of Shandong Qingdao Agricultural University Qingdao China; ^2^ Animal Husbandry General Station of Shandong Province Jinan China; ^3^ Urology Department Peking University Shenzhen Hospital Shenzhen China

**Keywords:** ceRNA network, Hippo signalling pathway, LH, pPGCLCs, proliferation

## Abstract

**Background:**

The transdifferentiation of skin‐derived stem cells (SDSCs) into primordial germ cell‐like cells (PGCLCs) is one of the major breakthroughs in the field of stem cells research in recent years. This technology provides a new theoretical basis for the treatment of human infertility. However, the transdifferentiation efficiency of SDSCs to PGCLCs is very low, and scientists are still exploring ways to improve this efficiency or promote the proliferation of PGCLCs. This study aims to investigate the molecular mechanism of luteinising hormone (LH) to enhance porcine PGCLCs (pPGCLCs) proliferation.

**Results:**

In this study, we dissected the proliferation regulatory network of pPGCLCs by whole transcriptome sequencing, and the results showed that the pituitary‐secreted reproductive hormone LH significantly promoted the proliferation of pPGCLCs. We combined whole transcriptome sequencing and related validation experiments to explore the mechanism of LH on the proliferation of pPGCLCs, and found that LH could affect the expression of Hippo signalling pathway‐related mRNAs, miRNAs and lncRNAs in pPGCLCs.

**Conclusions:**

For the first time, we found that LH promotes pPGCLCs proliferation through the competing endogenous RNA (ceRNA) regulatory networks and Hippo signalling pathway. This finding may help to elucidate the molecular mechanism by which LH promotes pPGCLCs proliferation.

## Graphical abstract

1. LH promotes the proliferation of pPGCLCs in vitro.
2. LH‐stimulated proliferation is accompanied by decreased apoptosis.
3. Hippo signalling pathway‐associated ceRNA networks played an important role in the proliferation of pPGCLCs.

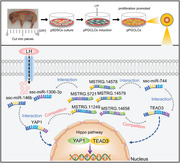

## 1 INTRODUCTION

With the rapid development of social industrialisation and environmental pollution, many high‐risk pathogenic factors now threaten human health. As a consequence, about 15% of couples worldwide suffer from infertility, and reproductive health problems seriously affect the normal quality of family life. In the face of this increasingly serious reproductive health situation, stem cell intervention for infertility treatment has gradually attracted more scientific and social attention.

Primordial germ cells (PGCs) are the earliest germ cells that produce male and female germ cells; therefore, they can serve as a good in vitro model for studying germ cell development.^1^, ^2^, ^3^ Researches in the past years have demonstrated that embryonic stem cells (ESCs) and/or induced pluripotent stem cells (iPSCs) derived from human, mouse and pig have the potential to differentiate into germ cells, oocyte‐like cells (OLCs) or sperm‐like cells,^4^, ^5^, ^6^, ^7^, ^8^, ^9^, ^10^, ^11^ which could develop to produce functional haploid gametes and even offspring after fertilisation.^9^, ^12^ However, this application is limited by the source of ESCs and the potential carcinogenicity of iPSCs. Recently, researchers have concentrated on inducing adult stem cells (ASCs) into OLCs in vitro owing to the advantages of more convenient collection and fewer ethical and moral constraints.^13^, ^14^ Some studies show that skin and germ cells are derived from the same embryonic germ layer, and some skin cells originate from the ectoderm; these cells are suggested to be the progenitor cells that maintain the pluripotency of the skin during early development.^15^, ^16^ Furthermore, some previous studies have shown that mouse SDSCs can be induced to differentiate into PGCLCs in vitro.^9^, ^17^, ^18^ In addition, the data of Ge et al. demonstrates for the first time that human foetal SDSCs have germline differentiation potential and can differentiate into germ cell‐like cells (GCLCs) in vitro under appropriate conditions.^8^

Despite success in both mice and humans, the induction of PGCLCs from pigs and other large animal pluripotent stem cells remains a great challenge. In 2006, Dyce et al. first obtained OLCs with zona pellucida structures from porcine SDSCs (pSDSCs) by culturing them in porcine follicular fluid.^7^ In 2009, Linher et al. obtained porcine PGCLCs (pPGCLCs) using pSDSCs and further induced them towards OLCs.^19^ However, the efficiency of SDSCs in generating PGCLCs is low at around 4%.^19^ This inefficiency of PGCLCs generation from stem cells is an important factor hindering mechanism research and high throughput screening. Previously, other studies have shown that some growth factors such as Midkine, BMP4, activin A (ActA) and retinoic acid (RA) can significantly promote the proliferation of pig or mouse PGCLCs.^20^, ^21^ Recently, data from Yan et al. show that RA regulates G1‐phase cell cycle progression through the ERK signalling pathway, thereby promoting the proliferation of pPGCLCs.^22^ Despite these findings, improvements in PGCLCs proliferation are still far from being understood.

Whole transcriptome sequencing is a frequently used method for understanding gene transcriptional information, which produces all transcripts in a sample, including mRNA and non‐coding RNA.^23^ The biological function of non‐coding RNAs has attracted much attention in recent years. As reported, only about 2% of the genome is transcribed into mRNAs, while a large amount of genomic DNA is transcribed into non‐coding RNAs.^24^, ^25^ MicroRNA (miRNA) is a small non‐coding RNA, consisting of 19–24 nucleotides that exist widely in eukaryotes.^26^ miRNA is involved in post‐transcriptional regulation, and it can bind multiple mRNAs in a way that is fundamentally complementary for regulating gene expression by inhibiting the translation of mRNA.^27^ Researches have shown that miRNAs play a pivotal role in the regulation of various organs and biological processes, such as cell growth and development, proliferation, apoptosis, and autophagy.^28^ Long non‐coding RNA (lncRNA) is another non‐coding RNA containing more than 200 nucleotides.^29^ As an important regulatory factor, lncRNA can affect various stages of gene transcription, modification, and translation, and is involved in the regulation of a broad range of biological processes, such as embryonic development, stem cell maintenance, cell differentiation and apoptosis.^30^, ^31^, ^32^ In 2011, Salmena et al. hypothesised the competing endogenous RNA (ceRNA), which suggests that lncRNAs can indirectly regulate the expression of protein‐coding genes by adsorbing miRNAs through miRNA‐responsive elements, thereby inhibiting the action of miRNAs.^33^ Currently, the regulatory networks of ceRNAs have become the focus of studying the roles of miRNA and lncRNA in cells.

Luteinising hormone (LH), secreted by the anterior pituitary basophils from puberty, is a glycoprotein hormone composed of two subunits. LH promotes the proliferation and development of follicular thecal cells to synthesise androgen, which provides the raw material for the synthesis of oestrogen by granulosa cells. The LH peak in the middle of menstruation is very important for follicular maturation and ovulation, and it also promotes the transformation of granulosa cells into follicular intima cells after ovulation.^34^ Furthermore, LH plays an important role in the acquisition of oocyte developmental potential.^35^, ^36^ LH and follicle‐stimulating hormone cooperate to maintain the development of pre‐ovulatory follicles to ovulation and luteal.^34^

In this study, the number of pPGCLCs was significantly increased after the administered of LH, and we hypothesised that LH could promote the proliferation of pPGCLCs. Therefore, in order to test our hypothesis, we performed whole‐transcriptome sequencing. Through data analysis and validation experiments, it was revealed that Hippo signalling pathway‐associated ceRNA networks played a vital role in the proliferation of pPGCLCs. Thus, this study provided a relatively clear and intuitive description of the molecular mechanism of LH‐enhanced pPGCLCs proliferation, which offers an important theoretical basis and research foundation for subsequent studies on PGCLCs.

## 2 MATERIALS AND METHODS

### 2.1 Sample collection and pSDSCs culture

Porcine foetuses were harvested from the Qingdao Wanfu Pig Breeding Base (Qingdao, Shandong, China). To avoid contamination, pig foetuses were transported to the laboratory on ice in the intact uterus. The pSDSCs culture method that was used in this experiment has been previously described.^22^, ^37^ Dorsal skin samples were taken from male or female pig foetuses on days 40–45 (E40‐45) of gestation. They were resected with a razor blade and washed with phosphate‐buffered saline (PBS). After washing, the skin was incubated with SDSC medium [Dulbecco's modified Eagle's medium (DMEM)‐F12 (1:1), 1% B‐27, 20 ng/ml epidermal growth factor (EGF) and 40 ng/ml basic fibroblast growth factor (bFGF)] in a 100 mm Petri dish (Sarstedt, Montreal, Canada). Four days later, when clusters of spherical cells appeared around the tissue, the tissue fragments were collected in 15 ml centrifuge tubes and the clusters were blown down with a pipette. The suspension of single porcine skin cells was incubated again with SDSC medium. After 24–48 h of incubation, the single cells had become aggregated into suspended skin spheres, and differentiation was induced by passing the suspension twice every 4 days.

### 2.2 Induction of pPGCLCs differentiation

After three passages, pSDSCs clone spheres were blown into individual cells at a density of 1 × 10^5^ cells per 60 mm dish (Corning, 430166, NY, USA). Then 3 ml of differentiation medium [DMEM, 5% foetal bovine serum (FBS; Gibco, Grand Island, NY, USA] and 5% porcine follicular fluid, 0.1 mM β‐mercaptoethanol) was added to each Petri dish that was subsequently incubated in a 37°C, 5% CO_2_ incubator. It took 48 h for all cells to adhere to the Petri dish and 8 days for them to cover the entire bottom of the Petri dish.

### 2.3 LH treatment

Cells were randomly divided into four Petri dishes: control, 200 mIU LH (Sigma, L5269) group, 400 mIU LH group, 600 mIU LH group. LH was added when cells were differentiated. Half of the medium was changed every 2 days, an appropriate amount of LH is added at each medium change to ensure that LH can fully play its role. LH was dissolved in DMEM and diluted to the desired concentration. The DMEM is our basic culture medium. So, there were no effects from the carrier and molecule. The control group was given appropriate DMEM according to the LH treatment group.

### 2.4 Immunofluorescence

After LH treatment, pPGCLCs were collected by centrifugation and fixed using 4% paraformaldehyde (PFA) overnight at 4°C. The cells were then smeared onto slides for subsequent experiments. After permeabilisation with PBST [PBS with 0.5% Triton‐X‐100 (Solarbio, Beijing, China)], the slides were blocked in TBST [10% goat serum in TBS (Boster, Wuhan, China)] for 35 min. The slides were then incubated with corresponding primary antibodies (Table S1) at an appropriate dilution. The next day, after washing three times, the slides were labelled with secondary antibodies (Table S1) at 37°C for 1 h. Nuclei were stained with Hoechst33342 (Beyotime, C1022,) for 5 min and representative pictures were captured using an Olympus BX51 fluorescence microscope imaging system (Olympus, Tokyo, Japan).

Image J software was used to determine the average fluorescence intensity per unit area. After immunofluorescence staining, at least five regions of interest (ROI) were selected on each slide to calculate the average fluorescence intensity per unit area. The fluorescence intensity was measured using the same parameters. After obtaining the average fluorescence intensity, we normalised the data with the control group as a reference to observe the change in fluorescence intensity in the treatment group.

### 2.5 Flow cytometry

The pPGCLCs were digested into a single cell suspension using 0.25% trypsin‐EDTA and subsequently fixed with 80% methanol. After the permeabilisation with 0.5% Triton‐X‐100, pPGCLCs were incubated with corresponding primary antibodies (Table S1) for 8 h at 4°C. Following a wash with PBS, pPGCLCs were incubated with secondary antibodies for 45 min at 37°C. After three further washes with PBS, pPGCLCs were analysed using a flow cytometer (Becton Dicksion, San Jose, CA, USA). The experiments were repeated at least three times and at least 10 000 cells were analysed in each group.

### 2.6 Western blotting (WB)

After washing, the collected pPGCLCs were lysed in RIPA lysis solution (Beyotime, P00113B) for 20 min on ice with vortexing. The extracted proteins were denatured by immersing in boiling water for 5 min and collected by centrifugation at 12 000 rpm for 3 min. The proteins were then separated by sodium dodecyl sulphate‐PAGE (SDS‐PAGE) and the proteins were electrophoretically transferred to poly‐vinylidene fluoride (PVDF) membranes (immobilon‐PSQ transfer membranes, Millipore, ISEQ00010, USA) with 200 mA for 150 min. After being blocked with 5% bovine serum albumin (BSA, Solarbio, A8020), the membranes were blocked with corresponding primary antibodies (Table S1) overnight. Following washes with TBST, membranes were incubated with secondary antibodies for 2 h (Table S1). Finally, the protein signalling was measured using a BeyoECL plus kit (Beyotime, P0018). GAPDH was selected as the housekeeping protein, and the data were analysed by AlphaView Sa Software (ProteinSimple, San Jose, CA, USA). The greyscales of the target proteins in different samples were analysed using the same area, and the greyscales of the target proteins were divided by that of the greyscales of the internal parameters to correct the errors. All the results were analysed without overexposure.

### 2.7 RNA Extraction from putative pPGCLCs and RT‐qPCR detection

Tissue total RNA extraction kit is based on Adelai's Easy Tissue/Cellular RNA Rapid Extraction Kit (Aidlab, RN28, Beijing, China). The sample was fully cleaved by the lysate and then transferred to a DNA scavenging column, where the filtrate was centrifuged and retained. After adding equal volume of ethanol, the filtrate was transferred to the adsorption column RA and centrifuged. Rinse twice with a rinse solution after removing proteins. After that, add RNase Free water, and the solution obtained was the RNA solution we needed. Use the RNA solution described above and then use Transgen's Transscript® One‐Step gDNA Removal and cDNA Synthesis Supermix box (TransGen, AT311‐03, Beijing, China) was used for reverse transcription of cDNA. The reactions were containing 1.0 μl anchored oligo(dT)18 primer (0.5 μg/μl), 10.0 μl 2× TS reaction mix, 1.0 μl TransScript® RT/RI enzyme mix, 1.0 μl gDNA remover and 7.0 μl RNA solution. The PCR conditions were as follows: 30 min at 42°C, 5 s at 85°C and 5 s at 4°C. The miRNA and lncRNA were reverse transcribed to cDNA using a Mir‐XTM miRNA First‐Strand Synthesis Kit (Takara, 638313, Dalian, China) and the PrimeScriptTM RT reagent Kit (Takara, RR047A), respectively. Table S2 shows the primers used for this experiment. Each sample contained three technical replicates and the relative mRNA expression levels of all genes were calculated by the formula of 2^−ΔΔCt^. GAPDH and U6 were used as housekeeping positive controls to normalise gene levels.

### 2.8 mRNA‐seq analysis

Whole transcriptome RNA sequencing was performed using a Novogene's Hiseq 4000 platform (Beijing, China). First, we checked the quality of the raw data using FastQC software and found low‐quality reads.^38^ Next, low‐quality sequences and remaining adapters were trimmed using fastp software.^39^ Subsequently, STAR Software was used to map reads to the *Sus scrofa* reference genome.^40^ We then sorted the resulting bam files using Samtools software and quantified transcript expression using featureCounts software.^41^ To reveal the differentially expressed mRNA, we analysed the quantitative results using the DESeq2 package.^42^ Later, we used ClusterProfiler software for gene set enrichment analysis and Cytoscape software for visualisation of Gene Ontology (GO) and Kyoto Encyclopedia of Genes and Genomes (KEGG) results.^43^, ^44^

### 2.9 miRNA‐seq analysis

The miRNA sequences were tested for quality in the same way as the mRNA sequences. We used Subread software and *Sus scrofa* reference miRNA from miRBase (http://www.mirbase.org/) for mapping.^45^ We then counted the mapping results using featureCounts software.^41^ Later, the DESeq2 package was also applied to identify differentially expressed miRNAs.^42^ To improve the accuracy of miRNA target gene prediction, we used TargetScan, RNAhybrid and miRWalk software for prediction.^46^, ^47^, ^48^, ^49^ Finally, we used clusterProfiler software for gene set enrichment analysis of target genes and Cytoscape software for visualisation of GO and KEGG results.^43^, ^44^

### 2.10 lncRNA‐seq analysis

lncRNA sequences were quality‐checked in the same way as mRNA sequences. We mapped reads to the *Sus scrofa* reference genome using STAR software and spliced transcripts using StringTie software.^40^, ^50^ We then merged all sample transcripts into one and compared them by using GffCompre software and reference annotation files.^51^ To accurately screen lncRNA, we first performed a preliminary screening based on the class_code type.^52^ Then, we used CPC2, CNCI and PfamScan software to identify lncRNA (Figure S7C).^53^, ^54^ Similarly, we used DESeq2 software to detect differentially expressed lncRNAs.^42^ Then, we used FEELnc (v 0.1.1) to find mRNAs cis‐regulated by lncRNAs.^55^ Finally, we used miRanda and RIsearch2 software to predict differentially expressed miRNAs (DEmiRNAs) that are targets of differentially expressed lncRNAs (DElncRNAs) based on the principle of base complementary pairing.^56^, ^57^

### 2.11 ceRNA network construction

Competing endogenous RNA (ceRNA) is a type of RNA that contains miRNA binding sites; it can competitively bind miRNA and inhibit miRNA's regulation of target genes. The ceRNA theory proposes that mRNA, pseudogenes, lncRNA, circRNA etc. may competitively bind miRNA through miRNA response element (MRE), thereby inhibiting the negative regulation of miRNA on target mRNA. We used the enrichment results of the signalling pathways of DEmRNAs and DEmiRNA target genes, and combined them with the DEmiRNAs targeted by DElncRNAs, to determine the signalling pathway with the greatest degree of regulation by the ceRNA network. We then determined the interaction regulation relationship among lncRNA‐miRNA‐mRNA based on the principle of a ceRNA network.^58^

### 2.12 Transfection of miRNA inhibitors

The inhibitors of ssc‐miR‐1306‐3p, ssc‐miR‐146b and ssc‐miR‐744 were synthesised at GenePharma (Jiangsu, China). We transfected in accordance with GenePharma's requirements. When the cell confluence rate reaches about 50–60%, the miRNA inhibitors were mixed with GP‐transfect‐Mate (GenePharma; Jiangsu, China), after settlement for 20 min, and add it to the cells culture medium. The cells were collected for subsequent experiments after 72 h.

### 2.13 Statistical analysis

The experimental verification in this study underwent at least three independent replicates and the bioinformatics analysis was repeated twice. All experimental results (expressed as mean ± SD) were analysed using GraphPad Prism 8 (GraphPad Software, San Diego, CA, USA). The values or percentages were analysed using one‐way analysis of variance (ANOVA) or Student's *t*‐test. Significant and highly significant differences are shown as *p < .05 and **p < .01.

## 3 RESULTS

### 3.1 LH exposure promotes the proliferation of pPGCLCs in vitro

**FIGURE 1:**
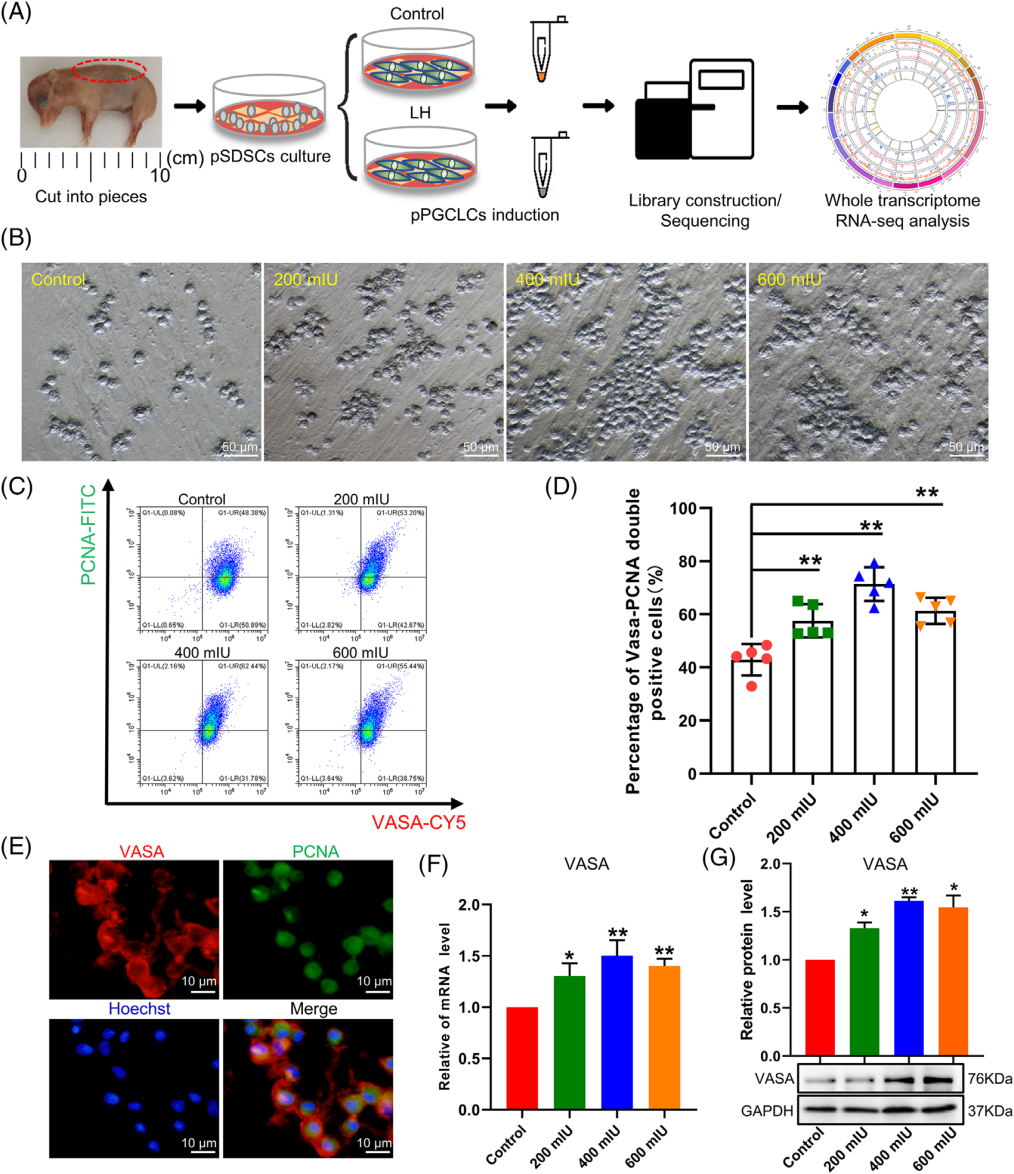
LH promotes the proliferation of pPGCLCs in vitro. (A) Schematic diagram of the research design. (B) Representative images of pPGCLCs treated with LH for 20 days. Bar = 50 μm. (C) Flow cytometry analysis of VASA and PCNA double‐positive pPGCLCs. (D) Statistics analysis of VASA and PCNA double‐positive pPGCLCs. (E) Immunofluorescent staining. Bar = 10 μm. (F) RT‐qPCR showing the mRNA level of VASA in pPGCLCs treated with different concentrations of LH. (G) WB showing the protein level of VASA in pPGCLCs treated with different concentrations of LH. The results of flow cytometry analysis were repeated five times and the results of Q‐PCR and WB were repeated at least three times. The results are presented as mean ± SD. *p < .05; **p < .01

To verify the role of LH in promoting the proliferation of pPGCLCs, we used an in vitro pPGCLCs induction differentiation model. The methods we used have been previously described.^22^ The entire experimental design involved in this study is presented in Figure 1A.

To localise the pSDSCs, we performed histological staining of foetal pigskin. We found that the hair follicle structure was not formed in the skin at this time, but the cells gathered to form the hair follicle precursors, and there were a large number of SOX2‐positive cells in the skin surface and the hair follicles precursor (Figure S1A). After 4 days of culture, many transparent cells were observed around the foetal pigskin, and these bright cells were identified as pSDSCs (Figure S1B and C). With the increase of subculture times, the clusters and structures of these clonal will become larger, and the edges of the clones became clearer and the structures became denser. Cell immunofluorescence showed that almost all cells were positive for stem cell‐specific markers SOX2, SOX9 and NANOG (Figure S1D). On the 16th day of differentiation induction of pSDSCs, there would be a large number of cells suspended (Figures 1B and S2A). These cells highly expressed the germ cell specific marker VASA, DAZL, PRDM14 and SOX17^10^, ^59^, ^60^ (Figures 1C and S2B).

In the effect detection section, we set up three LH concentration gradients (200, 400 and 600 mIU). The results showed that 400 mIU LH produced significantly more suspended individual or aggregated pPGCLCs on the 20th day of differentiation than the other treatment groups (Figure 1B). And we counted the number of pPGCLCs on the 20th day of differentiation and found that the number of 400 mIU LH‐treated cells was increased by 1.34‐fold (Figure S3A and B). The flow results are consistent with the counting results (Figure S3C). Afterwards, the 400 mIU LH‐treatment group had a significantly higher yield of VASA‐PCNA double positive pPGCLCs than the control groups, based on cell flow assay (1.67‐fold vs. control, *p* < .01; Figure 1C and D) and immunofluorescence assay (Figure 1E). In addition, we analysed the expression levels of the PGC marker VASA. As shown in Figure 1F and G, VASA mRNA level and protein expression were significantly elevated after the 400 mIU LH treatment compared with that of the control groups (*p *< .01). In combination with the above data, we chose 400 mIU LH for a follow‐up studies.

Next, we also examined genes related to proliferation and apoptosis such as CDK1, CDK2, CCND3, P21 and P53. We found that the expression of CDK1, CDK2 and CCND3 were significant increased and the expression of P21 and P53 were significant downregulated (Figure S4A). Furthermore, we also examined the expression of PCNA and found that PCNA were significantly upregulated both in RNA and protein levels (Figure S4B). Then we used EdU labelling and VASA co‐staining to further detect cell proliferation. After treatment with EdU kit, EdU‐VASA double positive cells were calculated by flow cytometry. The results showed that the EdU‐VASA positive cells increased from 39.3% to 62.5% in the LH‐treated group (Figure S4C). Moreover, we found that the pluripotency genes of pPGCLCs, such as NANOG and OCT4, were significantly increased, and SOX2 was significantly decreased after the addition of LH (Figure S4D). To determine whether LH treatment was connected with the apoptosis pathway, we analysed the expression of apoptosis‐related genes by WB. The results showed that Cleaved Caspase‐3 and BAX/BCL2 were significant decreased in the cells of LH‐treated (Figure S5A and B). This finding suggested that LH also suppress apoptosis of pPGCLCs.

In addition, we found that LH acted via LH receptor (LHR), and the expression of LHR was significantly increased in the 400 mIU LH treatment group (Figure S5C). Therefore, we concluded that LH has a mitogenic effect on pPGCLCs, mediated through promote cell proliferation as well as suppress apoptosis during in vitro induction via LHR.

### 3.2 Functional profiles of DEmRNAs

**FIGURE 2:**
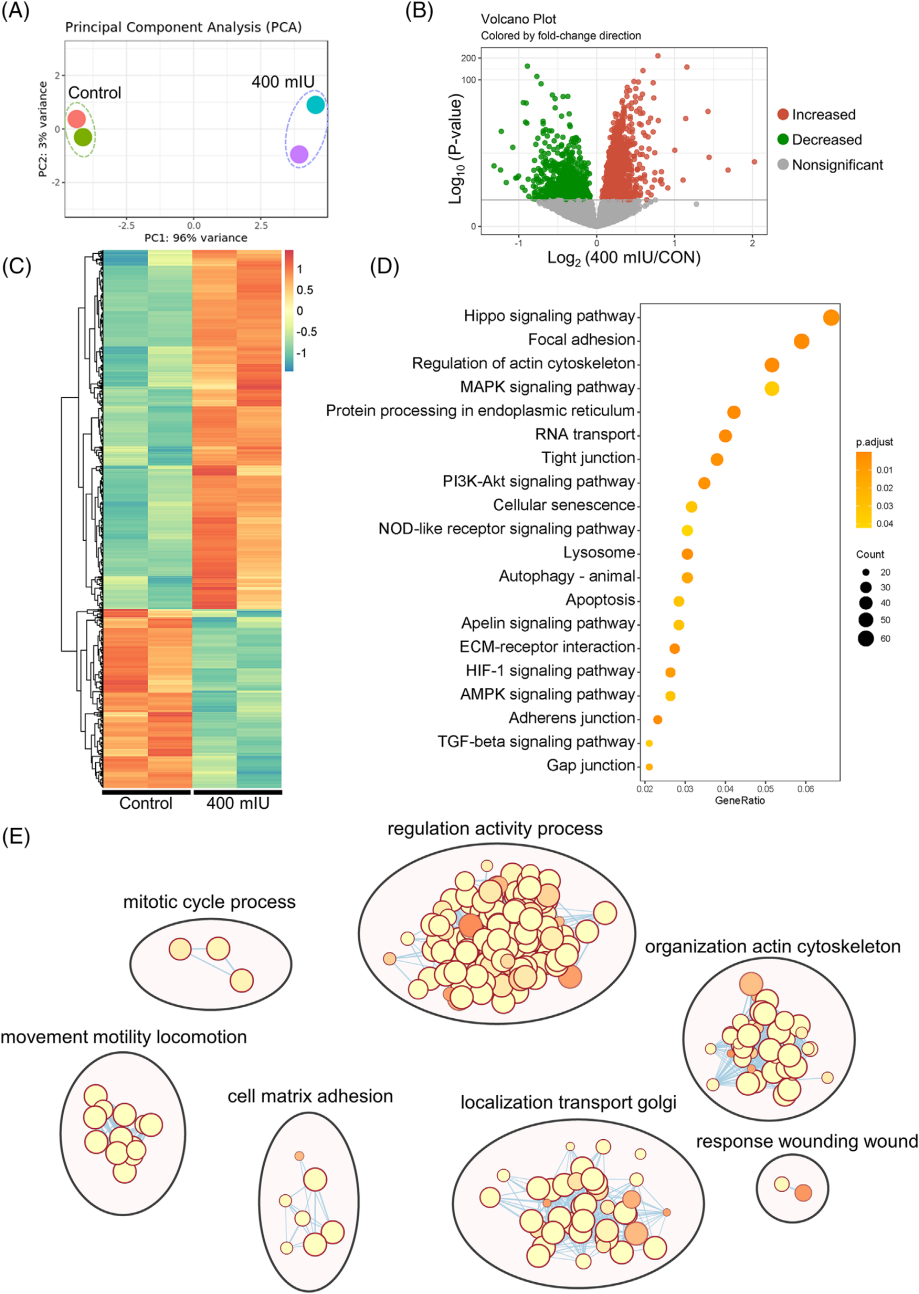
LH exposure alters the mRNA expression of pPGCLCs. (A) PCA analysis. (B) Volcano map demonstrating the DEmRNAs between the LH‐treatment and control groups. The green dots indicate that the DEmRNAs increased; the red dots indicate that the DEmRNAs decreased. (C) Heatmap demonstrating the expression model of DEmRNAs. From green to red gradient represents the expression levels from high to low. (D) KEGG enrichment analysis of DEmRNAs. (E) GO enrichment analysis of DEmRNAs

To further explore the molecular mechanism of LH in promoting the proliferation of pPGCLCs, we investigated the process using whole transcriptome sequencing. In this section, we first analytically portrayed the differential expression of mRNAs. Combining the expression levels of all mRNAs, we performed principal component (PCA) analysis. PCA showed that all repeats were clustered in the control and 400 mIU LH‐treated groups, respectively, and the difference between the control and treatment groups was statistically significant (Figure 2A). Subsequently, we screened differentially expressed mRNAs (DEmRNAs) between the different experimental groups. As shown in the volcano plot, we found that 1388 DEmRNAs were significantly increased and 690 DEmRNAs decreased in the 400 mIU LH‐treatment groups compared with the control groups (Figure 2B). Figure 2C further confirms that the clustering relationship of the samples is reliable, and shows the expression patterns of 2078 DEmRNAs in each group. It was also found that the expression of most genes in PGCLCs was significantly upregulated after LH treatment. It proves that these genes are indeed regulated by LH, which provides ideas for our next research. To better understand the role of these DEmRNAs, we performed KEGG and GO enrichment analyses. KEGG results showed that DEmRNAs were mainly focused on the signalling pathway of cell proliferation, metabolism and growth, similar to the Hippo signalling pathways and Focal adhesion (Figures 2D, E and S6A). Furthermore, we paid attention to the DEmRNAs enriched in the Hippo signalling pathway and found that most of them were affected by LH, and most of the gene expression levels are upregulated. Among them, the expression level of YAP1, TEAD3 and other genes were changed most significantly (Figure S6B).

### 3.3 LH treatment altered the expression of Hippo signalling pathway‐related miRNAs

**FIGURE 3:**
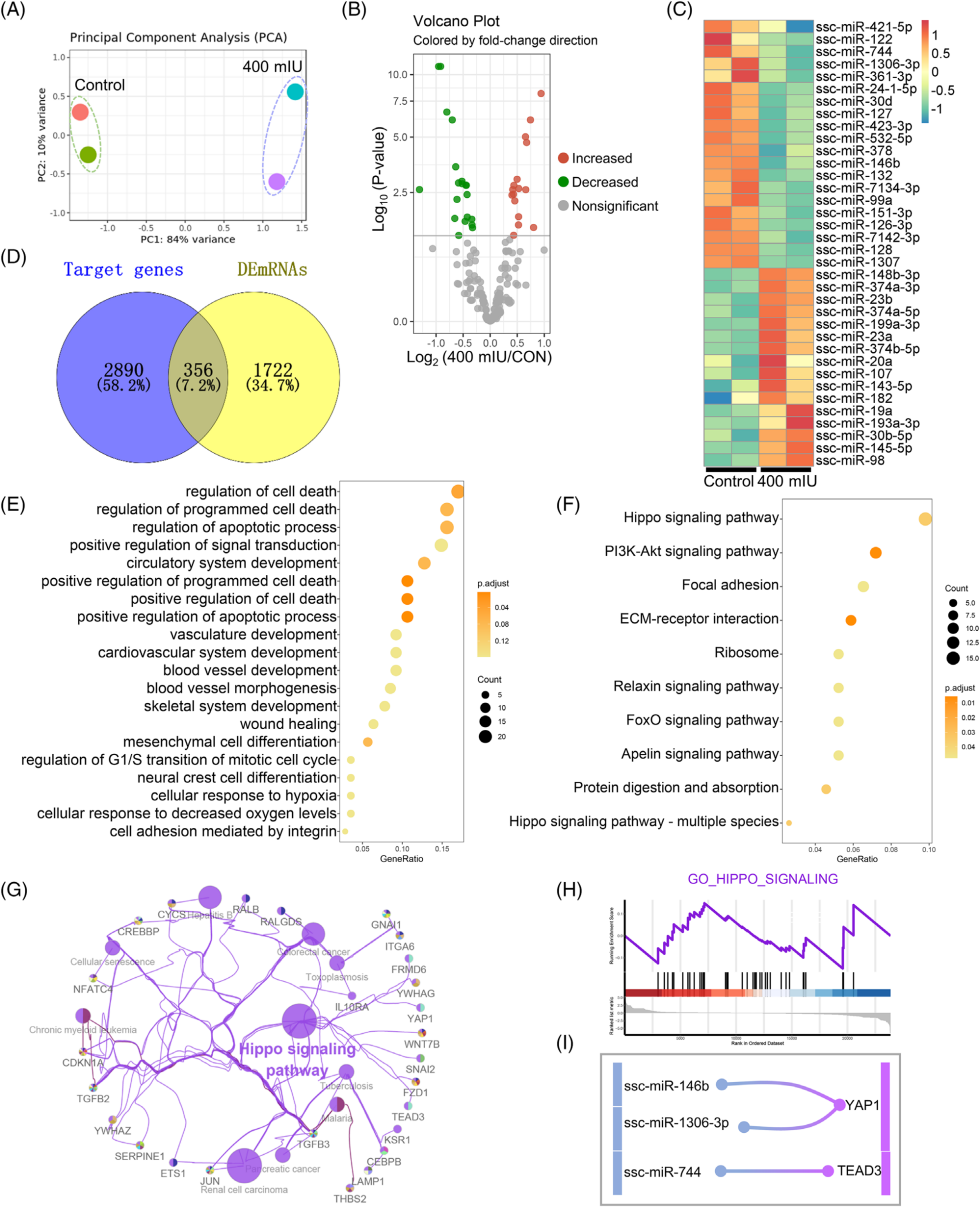
LH exposure alters the expression of miRNA in Hippo signalling pathway. (A) PCA analysis. (B) Volcano map demonstrating the DEmiRNAs between the LH‐treatment and control groups. The green dots indicate that the DEmRNAs increased; the red dots indicate that the DEmRNAs decreased. (C) Heatmap displaying the expression levels of DEmiRNAs. From green to red gradient represents the expression levels from high to low. (D) Venn diagram demonstrating the relationship between DEmRNAs and target genes. (E) The bubble chart demonstrating DEmiRNAs target genes GO enrichment results. (F) The bubble chart demonstrating DEmiRNAs target genes KEGG pathway enrichment results. (G) Enrichment map of KEGG pathway function of DEmiRNAs target genes. (H) Target genes of the miRNA enriched in the Hippo signal pathway via GSEA. (I) Sankey diagram of the miRNA‐mRNA network

In addition to the analysis of DEmRNAs, we also analysed relevant differentially expressed miRNAs (DEmiRNAs). We perform PCA analysis based on the expression of all miRNAs (Figure 3A). As shown in the volcano map, 16 DEmiRNAs were increased and 20 DEmiRNAs were decreased in the 400 mIU LH‐treatment group compared to the control (Figure 3B). Next, we used heatmaps to clarify placement of the DEmiRNAs in different groups (Figure 3C). We used RNAhybrid, miRanda and TargetScan software to predict the target genes of DEmiRNAs. Then, we selected the genes that overlap by the predicted target genes with DEmRNAs, and 356 genes (that is significantly differentially expressed mRNAs regulated by miRNAs) were produced (Figure 3D). Subsequently, we performed GO and KEGG enrichment analyses on the 356 target genes. Figure 3E shows that the enrichment of GO terms in biological processes was mainly associated with cell death, apoptosis, cell migration and regulation of cell differentiation. The same conclusion was reached for GO in terms of molecular function and cell composition (Figure S7A and B). Moreover, we carried out KEGG enrichment analysis of target genes of DEmiRNAs and mapped the top 10 pathways, including important pathways such as the Hippo signalling pathway, PI3K‐Akt signalling pathway and focal adhesion (Figure 3F; Table S4). Notably, the Hippo signalling pathway was significantly enriched and we constructed a network diagram of this pathway (Figure 3G). Gene Set Enrichment Analysis (GSEA) was used to analyse the function of DEmiRNAs, showed that Hippo signalling pathway‐related genes were increased in the LH‐treated group (Figure 3H). Furthermore, we found that YAP1 and TEAD3 were significantly upregulated in LH‐treated pPGCLCs (Table S3). These genes were the target genes of ssc‐miR‐146b, ssc‐miR‐744 and ssc‐miR‐1306‐3p, which were found to be decreased (Table S3). Correlation between the DEmRNAs and DEmiRNAs is shown in the Sankey plot in Figure 3I.

### 3.4 LH treatment altered the expression of lncRNAs

**FIGURE 4:**
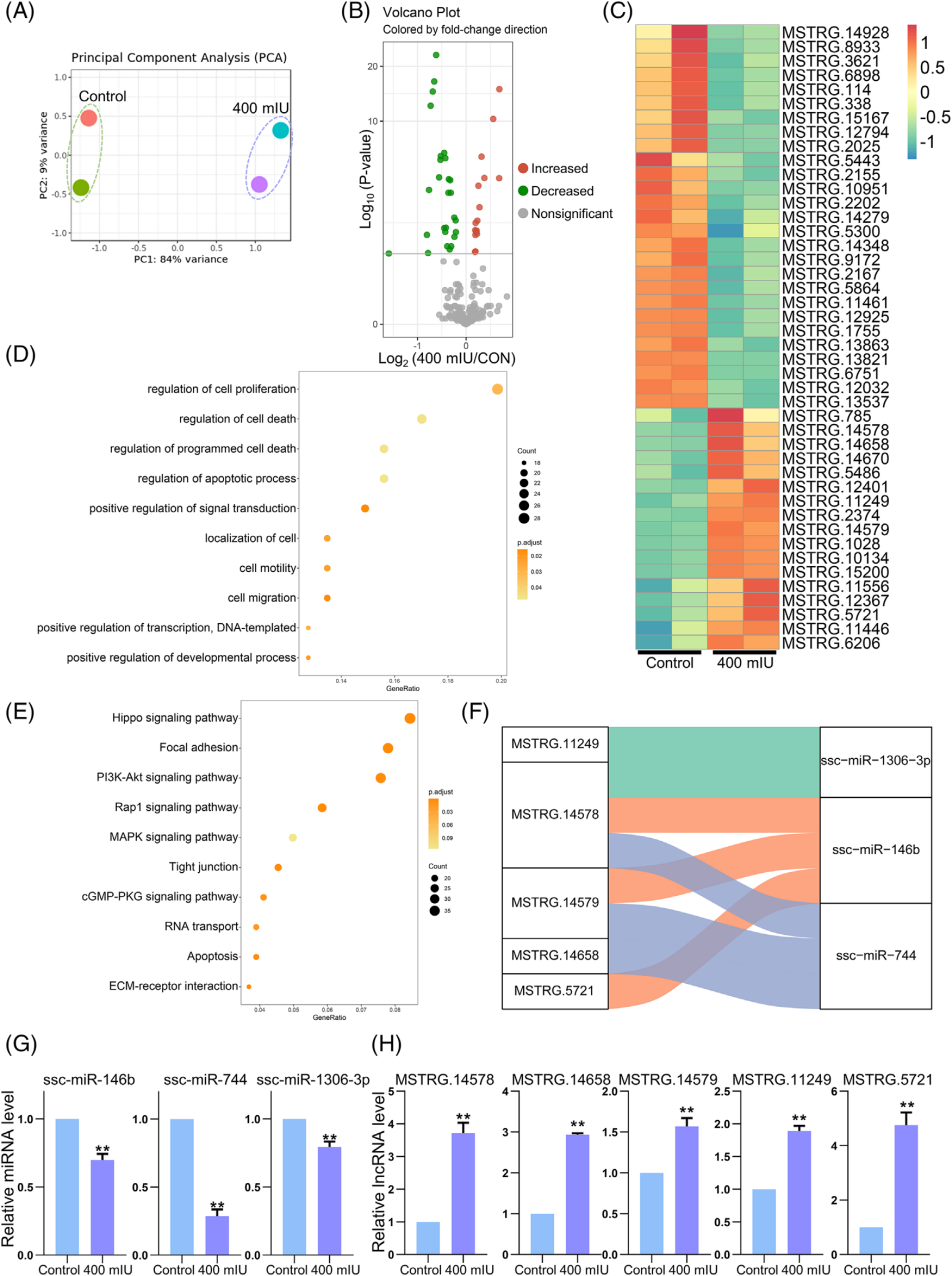
LH exposure alters the expression of lncRNA. (A) PCA analysis. (B) Volcano map demonstrating the DElncRNAs between the LH‐treatment and control groups. The green dots indicate that the DElncRNAs increased; the red dots indicate that the DElncRNAs decreased. (C) Heatmap displaying the expression levels of DElncRNAs. From green to red gradient represents the expression levels from high to low. (D) The bubble chart demonstrating DElncRNAs target genes GO enrichment results. (E) The bubble chart demonstrating DElncRNAs target genes KEGG pathway enrichment results. (F) Sankey diagram of the lncRNA‐miRNA network in pPGCLCs. Each rectangle represents a gene. (G) Analysis of the expression level of DEmRNAs related DEmiRNAs in pPGCLCs by RT‐qPCR. (H) Analysis of the expression level of DEmiRNAs related DElncRNAs in pPGCLCs by RT‐qPCR. All experiments were repeated at least three times. The results are presented as mean ± SD. *p < .05; **p < .01

After completing the analysis of differentially expressed miRNAs and mRNAs, we further analysed differentially expressed lncRNAs (DElncRNAs). PCA showed good clustering between the control and treatment groups (Figure 4A), while Volcano plots revealed the changes in DElncRNAs (Figure 4B). A total of 18 DElncRNAs were increased and 28 DElncRNAs were decreased in the 400 mIU LH‐treatment group (Figure 4B). The Heatmap in Figure 4C shows the differential expression pattern of these DElncRNAs. After that, we conducted GO enrichment analysis on the target genes of DElncRNAs and found that the genes were enriched to GO terms related to cell proliferation, cell death and cell motility (Figure 4D). And Hippo signalling pathway in the results of KEGG enrichment analysis still has important significance, which is consistent with the results of DEmRNAs (Figure 4E). Sankey diagram of the lncRNA‐miRNA network was then drawn to study the targeting relationship between DElncRNAs and DEmiRNAs (Figure 4F). RT‐qPCR results showed that the expression levels of ssc‐miR‐146b, ssc‐miR‐744 and ssc‐miR‐1306‐3p were significantly downregulated in the LH‐treated groups. In lncRNAs, the expressions of MSTRG.14578, MSTRG.14658, MSTRG.14579, MSTRG.11249 and MSTRG.5721 were significantly upregulated (*p* < .01; Figure 4G and H).

### 3.5 Validation of YAP1and TEAD3 association with Hippo signalling pathways

**FIGURE 5:**
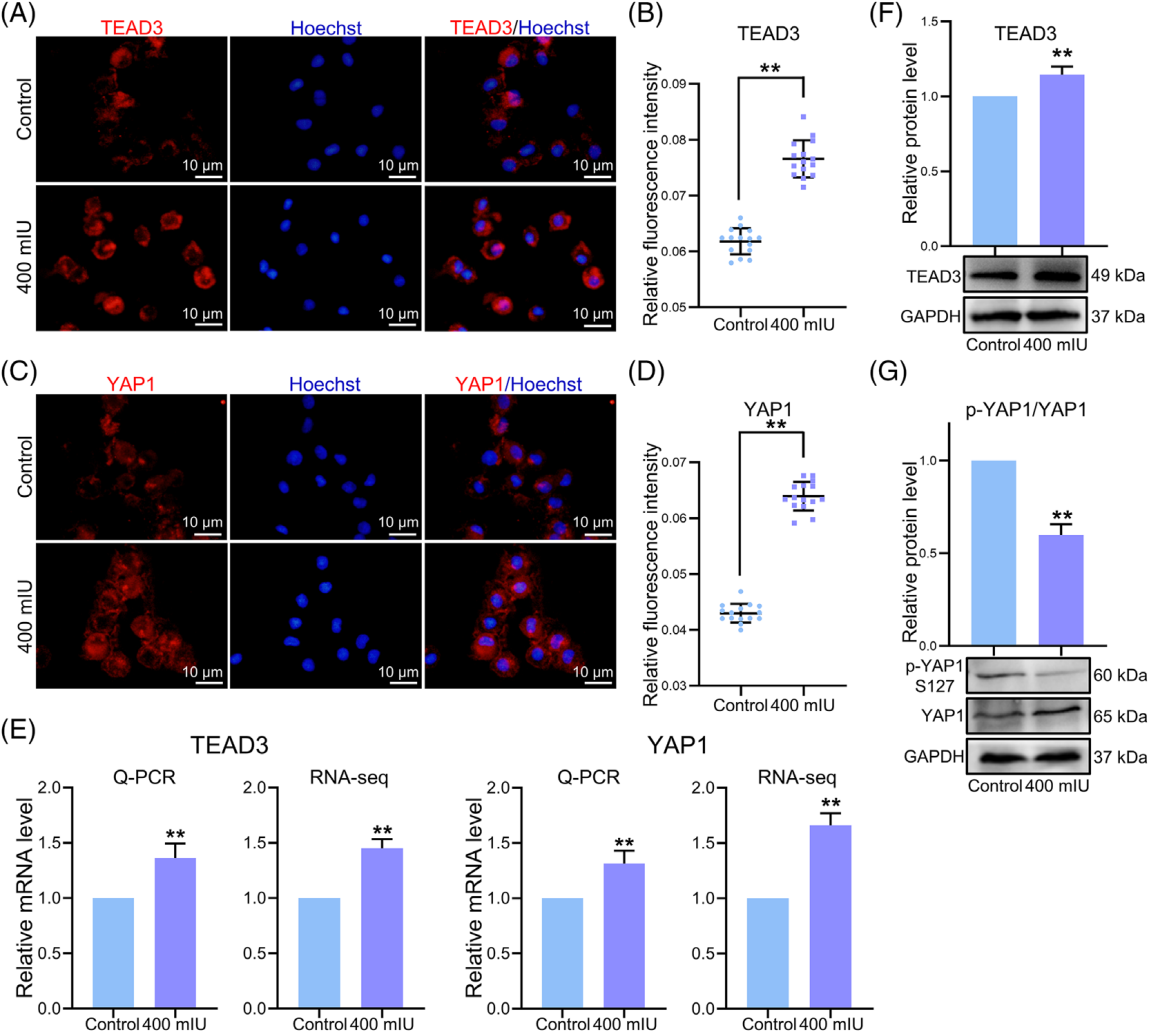
Verification of the expression of TEAD3 and YAP1. (A) Immunostaining of TEAD3 (red) in pPGCLCs; Hoechst 33342 (blue) was used for nuclei staining. (B) The analysis of the relative fluorescence intensity of TEAD3. (C) Immunostaining of YAP1 (red) in pPGCLCs; Hoechst 33342 (blue) was used for nuclei staining. (D) The analysis of the relative fluorescence intensity of YAP1. (E) Analysis of the expression level of TEAD3 and YAP1 in pPGCLCs by RT‐qPCR and RNA‐sEquation (F, G) Analysis of the expression level of TEAD3 and p‐YAP1/YAP1in pPGCLCs by WB. All experiments were repeated at least three times. The results are presented as mean ± SD. *p < .05; **p < .01

We carried out cell immunofluorescence, WB and RT‐qPCR to analyse their expression to further verify effect of LH treatment on changes in YAP1 and TEAD3. In the LH treatment groups, the fluorescence intensity of TEAD3 was significantly increased compared with the control (Control = 0.0618 ± 0.0023; 400 mIU = 0.0766 ± 0.0033, *p *< .01; Figure 5A and B). Moreover, the fluorescence intensity of YAP1 had the same tendency (Control = 0.0430 ± 0.0017; 400 mIU = 0.0639 ± 0.0026, *p *< .01; Figure 5C and D). In addition, we examined the expression of YAP1 and TEAD3 (Figure 5E), which were significantly upregulated at the mRNA level (*p *< .01) and the protein level of TEAD3 was also increased (Figure 5F). The phosphorylation of YAP1 (p‐YAP1) could inhibit YAP1 activity, so we also tested the expression of p‐YAP1 on S127. As shown in Figure 5G, WB showed that YAP1 in the LH treatment group was increased and the expression of p‐YAP1 was significantly decreased (*p *< .01), indicating that unphosphorylated YAP1 was entered the nucleus to play a role in transcriptional assistant activity.

### 3.6 Hippo signalling pathway‐related ceRNA network

**FIGURE 6:**
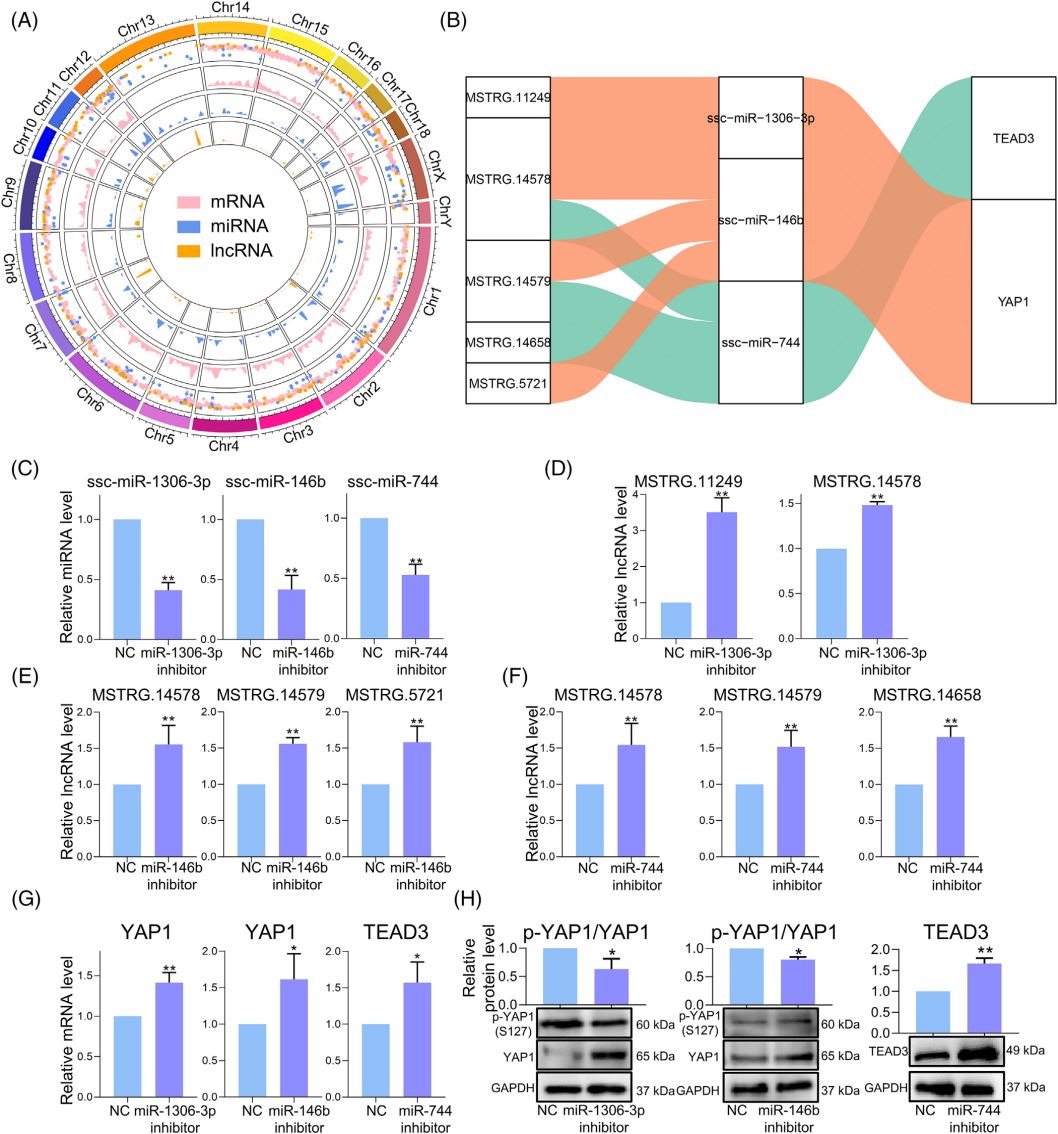
The lncRNA‐miRNA‐mRNA ceRNA network. (A) Genome‐wide distribution of DEmRNAs, DEmiRNAs and DElncRNAs. (B) Sankey diagram of the lncRNA‐miRNA‐mRNA ceRNA network in pPGCLCs. Each rectangle represents a gene. (C) The expression levels of miR‐1306‐3p, miR‐146b and miR‐744 after miRNA inhibitor treatment. (D) The expression level of target lncRNAs after miR‐1306‐3p inhibitor treatment. (E) The expression level of target lncRNA after miR‐146b inhibitor treatment. (F) The expression level of target lncRNA after miR‐744 inhibitor treatment. (G) The expression levels of YAP1 and TEAD3 after miRNA inhibitor treatment. (H) The protein expression levels of YAP1 and TEAD3 after miRNA inhibitor treatment. The results are presented as mean ± SD. *p < .05; **p < .01

We displayed the gene expression and distribution on chromosomes of DEmRNAs, DEmiRNAs and DElncRNAs on a Chord Diagram and found that the distributions were different. This revealed that ncRNA plays an indispensable role in regulating mRNA, which also demonstrates that ncRNA plays a vital role in the regulation of LH on pPGCLCs (Figure 6A). To gain further insights into the mechanism of LH regulating the Hippo signalling pathway through ncRNA, we constructed and analysed the regulatory network of ceRNAs based on mRNA‐miRNA, mRNA‐lncRNA and miRNA‐lncRNA target pairs (Figure 6B). And by observing the binding sequence of each RNAs in the ceRNA network, it is found that the binding sequence between all targets is very strong (Figure S8A and B). In addition, we used the method of transfecting miRNA inhibitors to downregulate the expression levels of ssc‐miR‐146b, ssc‐miR‐744 and ssc‐miR‐1306‐3p (Figure 6C). The results of RT‐qPCR showed that compared with the negative control group, the expression of MSTRG.14578, MSTRG.14658, MSTRG.14579, MSTRG.11249, MSTRG.5721, YAP1 and TEAD3 were significantly upregulated in the miRNA inhibitors treatment group (Figure 6D–G). Later, we found that at the protein level, the expression of YAP1 and TEAD3 was also significantly upregulated compared with the negative control group (Figure 6H). This proves the reliability of our results.

## 4 DISCUSSION

In this study, we induced pPGCLCs using a platform established in our laboratory and performed detailed analysis of mRNA, miRNA and lncRNA in combination with whole transcriptome sequencing. From the whole transcriptome sequencing results, we found that LH promoted the proliferation of pPGCLCs, at least in part, because LH regulated the lncRNA‐miRNA‐mRNA ceRNA network, which in turn affected the Hippo signalling pathway. Taken together, these results help us to better comprehend the role of LH in pPGCLCs proliferation through the ceRNA network‐mediated Hippo pathway.

Research addressing stem cell‐induced differentiation into germ cells has recently received increasing attention. Currently, many types of stem cells have been found to have the potential to be induced into germ cells in vitro.^9^, ^20^, ^61^, ^62^, ^63^, ^64^ It is well known that the quantity and quality of PGCLCs derived from stem cells are critical for differentiation towards gametes in vitro. However, the current efficiency of obtaining germ cells from stem cells is still very low, which indicates the lack of a substance that effectively increases the number of PGCLCs in vitro. In recent years, numerous studies have demonstrated that small molecules have amazing potential and advantages in reprogramming cellular fate.^65^, ^66^ In 2015, Sun et al. found that Act A can effectively increase the efficiency of differentiating mouse SDSCs into PGCLCs in vitro and found that Smad3 plays a key regulatory role in the pathway of Ac A action.^17^ In 2016, Tan et al. found that RA can promote the expression of cell cycle‐related genes and regulate cell cycle processes, thereby promoting the proliferation of SDSCs‐derived PGCLCs in mice.^18^ The endogenous hormone LH plays an important role in promoting maturation of the reproductive system.^67^ However, to date, there are few reports on the effects of LH on stem cell differentiation. By adding different concentrations of LH to culture media, we found that the number of pPGCLCs was significantly higher in the 400 mIU LH group. Moreover, flow cytometry analysis showed that the number of VASA‐PCNA double‐positive cells was significantly higher than that of other groups. In addition, the results showed that VASA‐positive cells were also PCNA‐positive cells. Therefore, we suggest that LH promotes the proliferation of pPGCLCs in the process of differentiation.

The transcription level of mRNA is affected by ncRNA regulation. In analysing the entire biomolecular network and regulatory pathways, it is necessary to quantitatively and qualitatively study all RNA molecules in the whole transcriptome. Whole transcriptome sequencing allows complete annotation and quantification of all genes and their subtypes in a sample, giving a full and true depiction of the sample transcripts.^68^, ^69^ More importantly, whole transcriptome sequencing can more directly and clearly predict the functional relationship between ncRNAs and their target genes. Here, we combined the results of bioinformatics analysis with experimental results to elucidate the mechanism by which LH promotes the proliferation of pPGCLCs. Given the difficulty in collection of our samples, there were only two replicates in the transcriptome sequencing. In order to improve the accuracy of our data, we collected the dorsal skins of pig foetuses from four sows respectively as sequencing samples. Although there were only two replicates, the experimental verification was highly consistent with the transcriptome sequencing, which ensured the reliability of the data. In this study, we obtained DEmRNAs, DEmiRNAs and DElncRNAs from the control and treatment groups. We further analysed the pathways and functions of these differentially expressed genes using GO and KEGG analyses. The results indicated that some biological process GO terms were associated with the Hippo signalling pathway, which were also enriched at the mRNA and miRNA levels. Many studies have focused on the overall network regulation of ncRNAs rather than on individual molecules. There are various regulatory mechanisms between lncRNAs and miRNAs. lncRNAs can regulate the expression of miRNAs, and conversely, miRNAs can also regulate the expression of lncRNAs.^33^ In‐depth analysis of the ceRNA network has extended our understanding of coding and non‐coding RNA function. Thus, we determined lncRNAs by predicting the target genes of miRNAs and constructed a proliferation‐associated ceRNA network (Figure 6B). Here, we investigated the correlation of five lncRNAs, three miRNAs and two mRNAs to more clearly visualise the mechanism by which LH regulates the proliferation of pPGCLCs. Researches have shown that miRNA plays a pivotal role in promoting the proliferation of stem cells and inducing the directed differentiation of stem cells.^70^ We found that ssc‐miR‐146b, ssc‐miR‐744 and ssc‐miR‐1306‐3p were all downregulated after the addition of LH. Among them, miR‐146b was found to be closely related to the proliferation and apoptosis of bovine male germ stem cells, miR‐744 was shown to be related to cell proliferation, cell cycle and cell growth, and our results were consistent with these results.^71^, ^72^, ^73^ The downregulation of miRNA leads to the upregulation of the corresponding five lncRNAs, which then activates the Hippo signalling pathway closely related to proliferation.

The Hippo signalling pathway has been extensively studied in mammalian cells and animal models.^74^, ^75^, ^76^, ^77^ YAP1 (yes‐associated protein 1), the main effector of the Hippo pathway, remains silent in the normal organism and maintains a stable balance of cell proliferation and differentiation.^78^ Numerous researches showed that the Hippo signalling pathway plays a pivotal role in determining the fate of stem or progenitor cells. Activated YAP1 has been reported to induce cyclin D1 and inhibit the expression of NeuroM, thereby accelerating the cycle process, shortening the cycle length and regulating the proliferation efficiency of neural stem cells.^79^ In addition, Schlegelmilch et al. have found that YAP1 protein can regulate the proliferation of epidermal stem cells.^80^ The regulation of cell proliferation and tissue growth takes place through initiation of the transcription of downstream genes, mainly through binding to TEAD family transcription factors. TEAD family transcription factors are major partners in the biological function of YAP1. The protein complex formed between TEAD and YAP1 plays a vital role in YAP1‐mediated cell proliferation.^81^, ^82^, ^83^, ^84^ In our study, the expression level of YAP1 was significantly in LH‐treated pPGCLCs compared to the control group. After activation, YAP was further transcribed in combination with TEAD3. So TEAD3 was also significantly upregulated both at gene and protein levels. A previous study found that phosphorylation of YAP1, particularly at the S127 site, regulates YAP1 localisation and degradation.^85^ The phosphorylated YAP1 combines with 14‐3‐3 protein and blocks in the cytoplasm and inactivated. The unphosphorylated YAP enters the nucleus and binds with the transcriptional activator TEAD3 to jointly initiate the transcription of downstream genes, thus playing the role of transcriptional auxiliary activity.^86^, ^87^ In our experiment, we detected the expression of YAP1 and p‐YAP1, and we found that the expression of YAP1 in the LH treatment group was increased and the expression of p‐YAP1 was significantly decreased, so we believed that the Hippo signalling pathway was activated.

To our knowledge, this is the first study to describe the effect of LH on the proliferation of pPGCLCs. Our data showed that LH exposure could affect mRNA and ncRNA expression profiles of pPGCLCs, and we enriched the Hippo signalling pathway by constructing a ceRNA regulatory network. In conclusion, this study provides support for our discovery that LH promotes the proliferation of pPGCLCs through the ceRNA network mechanism and the Hippo signalling pathway.

## CONFLICT OF INTERESTS

The authors have no competing financial interests to declare.

## Supporting information


**Table S1**. Antibodies used in this paper
**Table S2**. Primers Used for Quantitative RT‐PCR
**Table S3**. Genes expression in lncRNA‐miRNA‐mRNA network
**Table S4**. Analysis of KEGG pathways of target genes (356)

**Figure S1. The characterisation of pSDSCs**. (A) The expression of SOX2 in the pig foetuses skin. Bar = 50 μm. (B) Morphology of pig foetuses skins at different days. Bar = 50 μm. (C) Colony morphology of pSDSCs at different passages. Bar = 50 μm. (D) SOX2, SOX9 and NANOG immunocytochemistry of pSDSCs. Bar = 50 μm

**Figure S2. The characterisation of pPGCLCs**. (A) Different days of pPGCLCs. Bar = 50 μm. (B) DAZL, PRDM14 and SOX17 immunocytochemistry of pPGCLCs. Bar = 50 μm

**Figure S3. The number of pPGCLCs increased significantly after the addition of LH**. (A) Cell images of the control and LH‐treated group at 0 and 20 days. (B) Number of cells cultured for 20 days of control and LH‐treated group. (C) Flow cytometry analysis of VASA positive pPGCLCs. The results are presented as mean ± SD. *p < .05; **p < .01

**Figure S4. LH promotes the proliferation of pPGCLCs**. (A) Effect of LH on the levels of mRNA of proliferation genes. (B) The expression of PCNA. (C) Flow cytometry analysis of VASA and EdU double‐positive pPGCLCs. (D) Change in pluripotency of pPGCLCs. The results are presented as mean ± SD. *p < .05; **p < .01

**Figure S5. LH‐stimulated proliferation is accompanied by decreased apoptosis**. (A) WB images of BAX, BCL2, Caspase‐3 and Cleaved Caspase‐3. (B) The relative protein level of Cleaved Caspase‐3 and BAX/BCL2. (C) The expression of LHR. The results are presented as mean ± SD. *p < .05; **p < .01

**Figure S6. GO enrichment results (RNA‐seq)**. (A) GO Biological Process. (B) Heatmap of DEmRNAs in Hippo signalling pathway

**Figure S7. GO term of DEmiRNA and identification of candidate lncRNA**. (A) GO Molecular Function. (B) GO Cellular Component. (C) The Venn diagram showing the selected lncRNAs

**Figure S8. Schematic diagram of binding sequence**. (A) Schematic diagram of the binding sequence of miRNAs and lncRNAs. (B) Schematic diagram of the binding sequence of miRNAs and mRNAs
