# Supplementary material for: The proliferation role of LH on porcine primordial germ cell‐like cells (pPGCLCs) through ceRNA network construction
Source: Clin Transl Med. 2021 Oct 14;11(10):e560. doi: 10.1002/ctm2.560 (PMC8516341; doi:10.1002/ctm2.560)
Supplement: Supplementary file 1 — Table S1. Antibodies used in this paper Table S2. Primers Used for Quantitative RT‐PCR Table S3. Genes expression in lncRNA‐miRNA‐mRNA network Table S4. Analysis of KEGG pathways of target genes (356) [file CTM2-11-e560-s005.docx]

**Supporting Information**

**
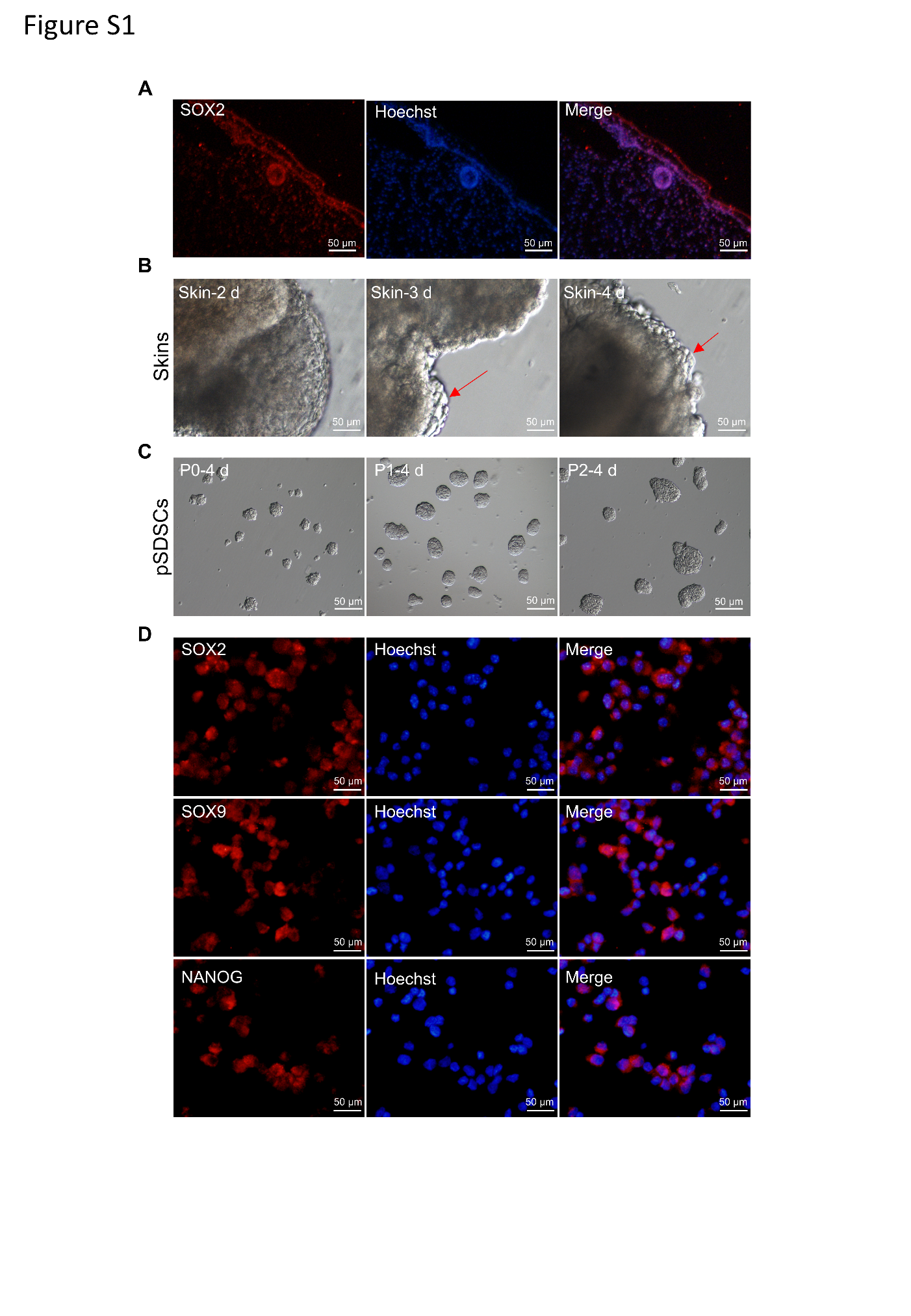
**

**Figure S1. The characterization of pSDSCs.** (A) The expression of SOX2 in the pig fetuses skin. Bar = 50 μm. (B) Morphology of pig fetuses skins at different days. Bar = 50 μm. (C) Colony morphology of pSDSCs at different passages. Bar = 50 μm. (D) SOX2, SOX9 and NANOG immunocytochemistry of pSDSCs. Bar = 50 μm.

**
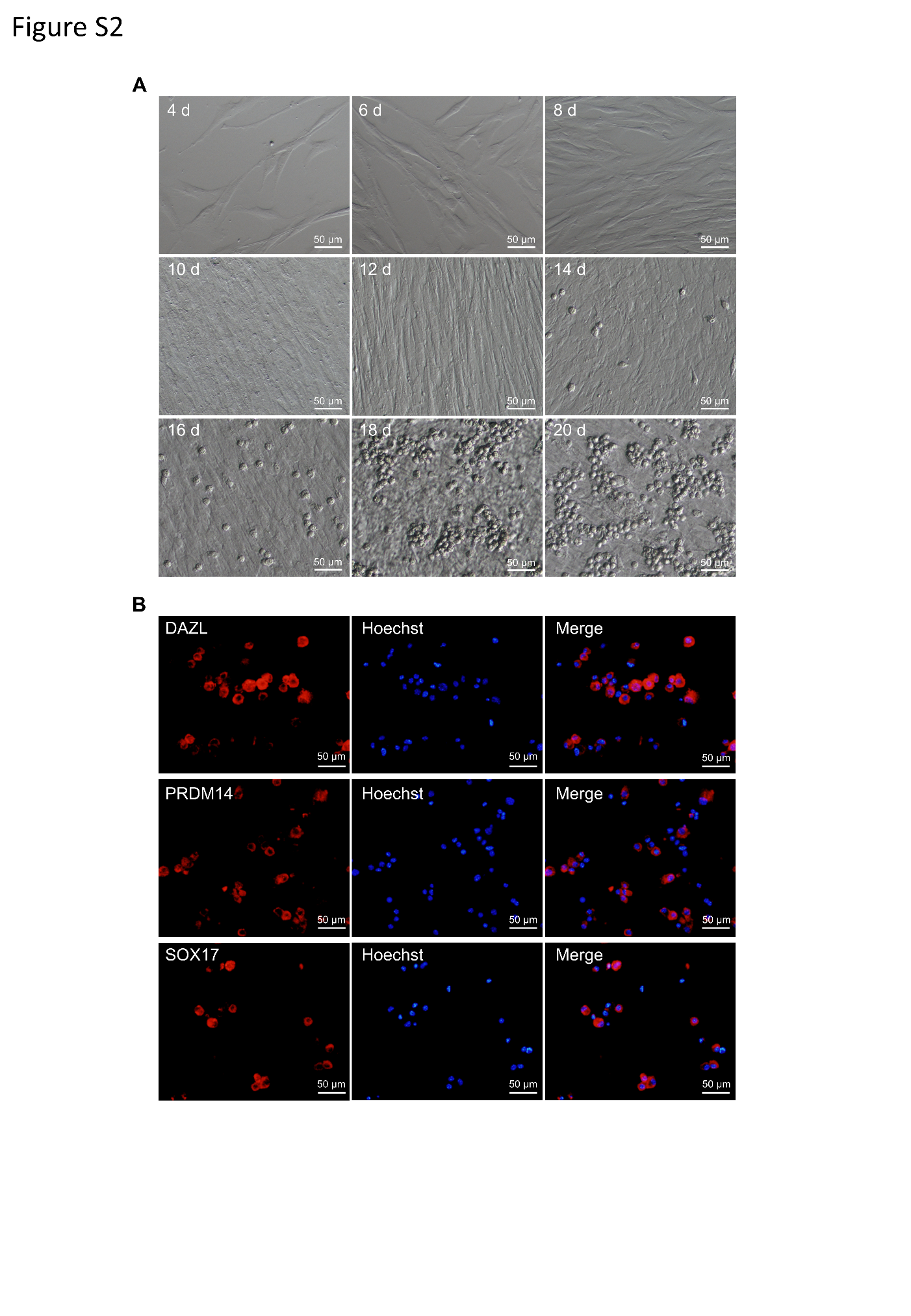
**

**Figure S2. The characterization of pPGCLCs.** (A) Different days of pPGCLCs. Bar = 50 μm. (B) DAZL, PRDM14 and SOX17 immunocytochemistry of pPGCLCs. Bar = 50 μm.

**
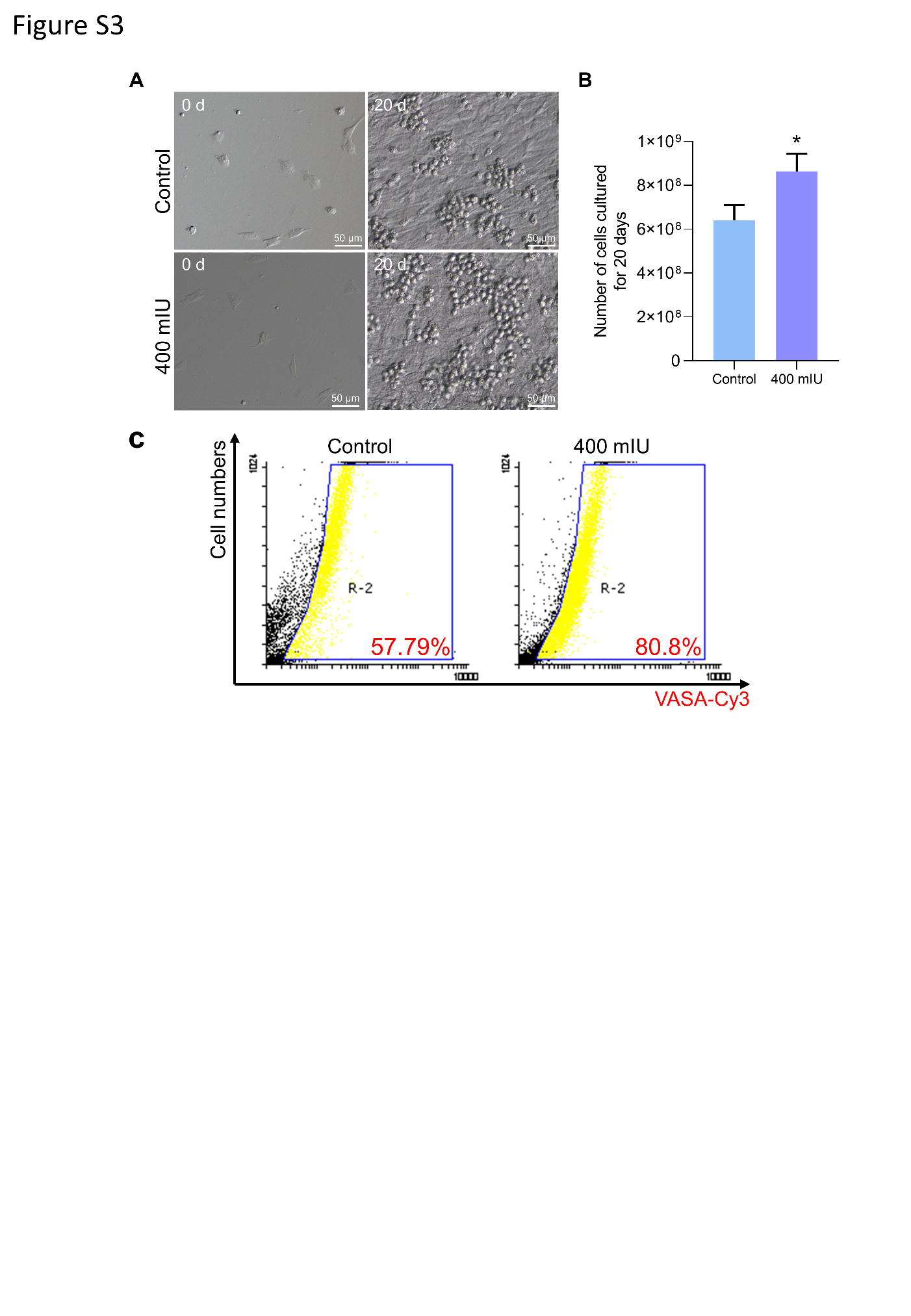
**

**Figure S3.** **The number of pPGCLCs increased significantly after the addition of LH.** (A) Cell images of the control and LH-treated group at 0 d and 20 d. (B) Number of cells cultured for 20 d of control and LH-treated group. (C) Flow cytometry analysis of VASA positive pPGCLCs. The results are presented as mean ± SD. **P* < 0.05; ***P* < 0.01.

**
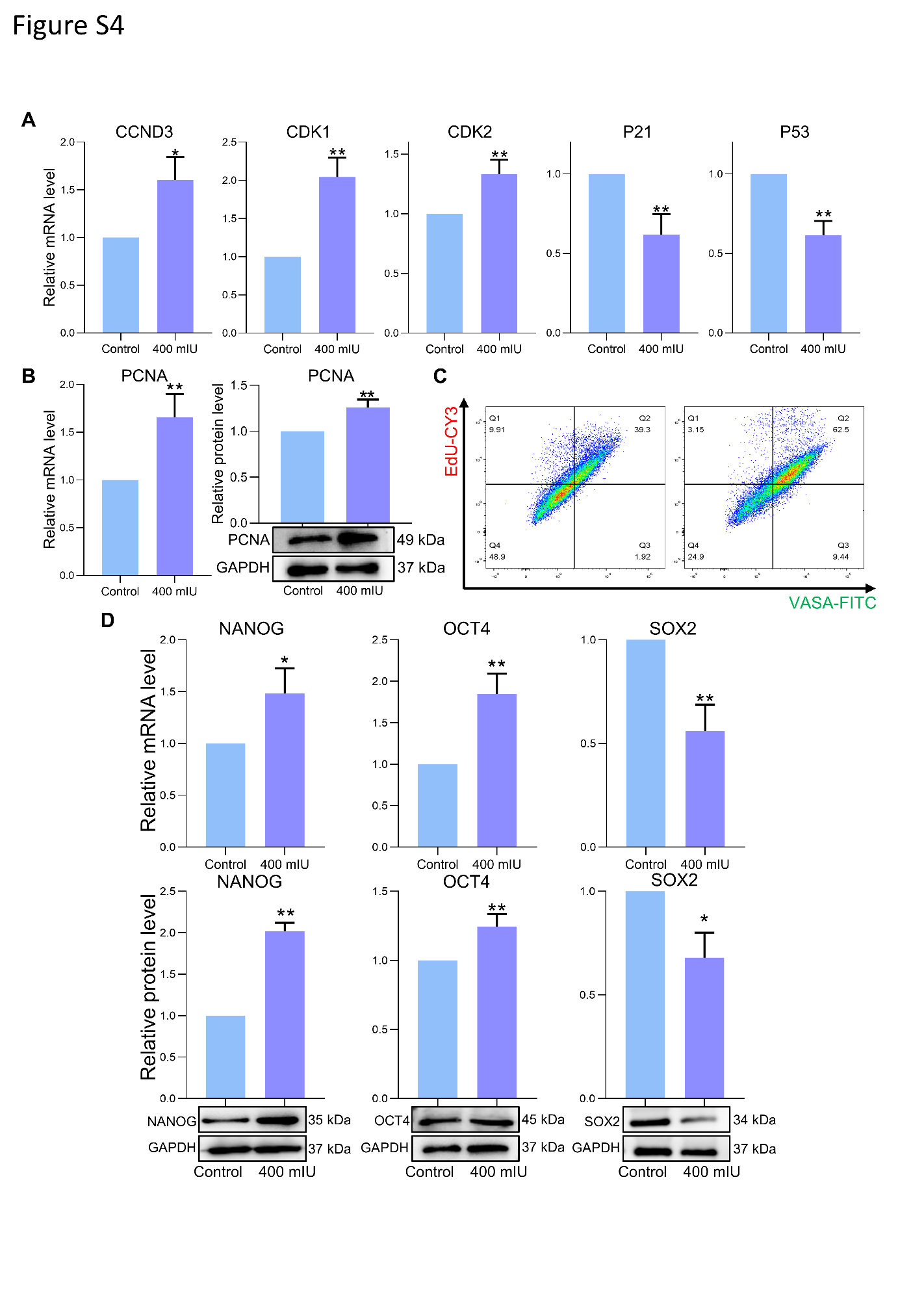
**

**Figure S4. LH promotes the proliferation of pPGCLCs.** (A) Effect of LH on the levels of mRNA of proliferation genes. (B) The expression of PCNA. (C) Flow cytometry analysis of VASA and EdU double‐positive pPGCLCs. (D) Change in pluripotency of pPGCLCs. The results are presented as mean ± SD. **P* < 0.05; ***P* < 0.01.

**
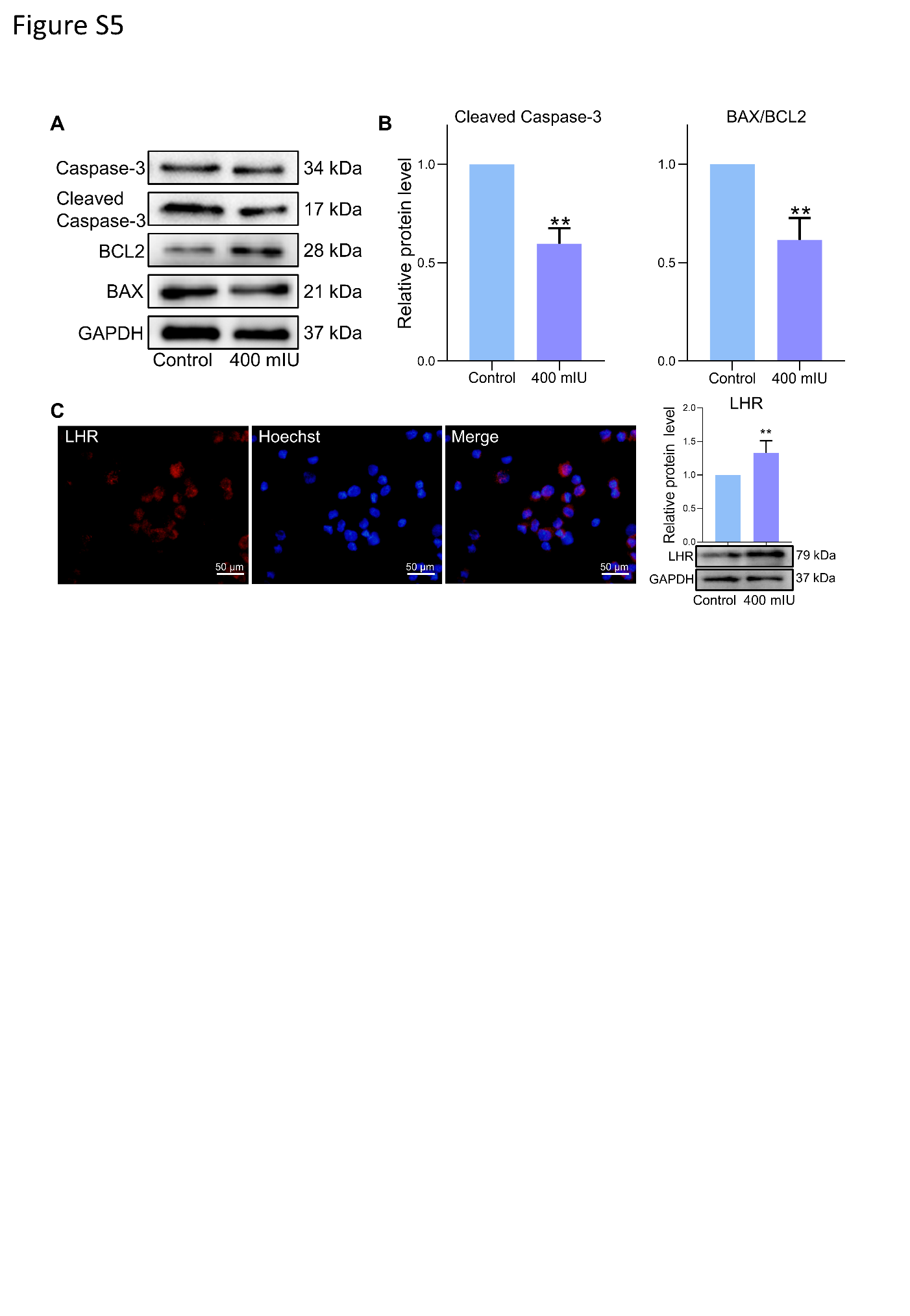
**

**Figure S5. LH-stimulated proliferation is accompanied by decreased apoptosis.** (A) WB images of BAX, BCL2, Caspase-3 and Cleaved Caspase-3. (B) The relative protein level of Cleaved Caspase-3 and BAX/BCL2. (C) The expression of LHR. The results are presented as mean ± SD. **P* < 0.05; ***P* < 0.01.

**
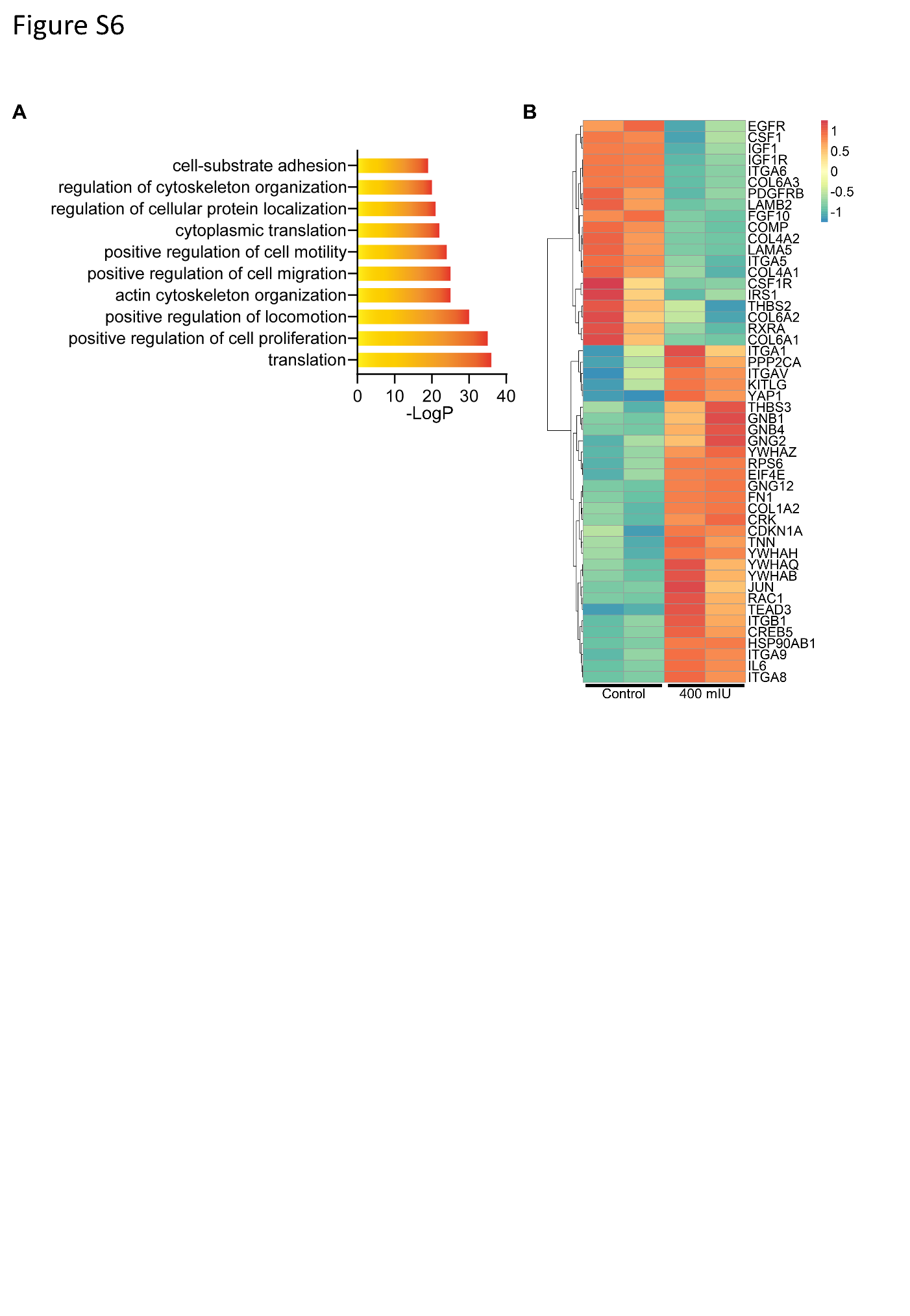
**

**Figure S6. GO enrichment results (RNA-seq).** (A) GO Biological Process. (B) Heatmap of DEmRNAs in Hippo signaling pathway.

**
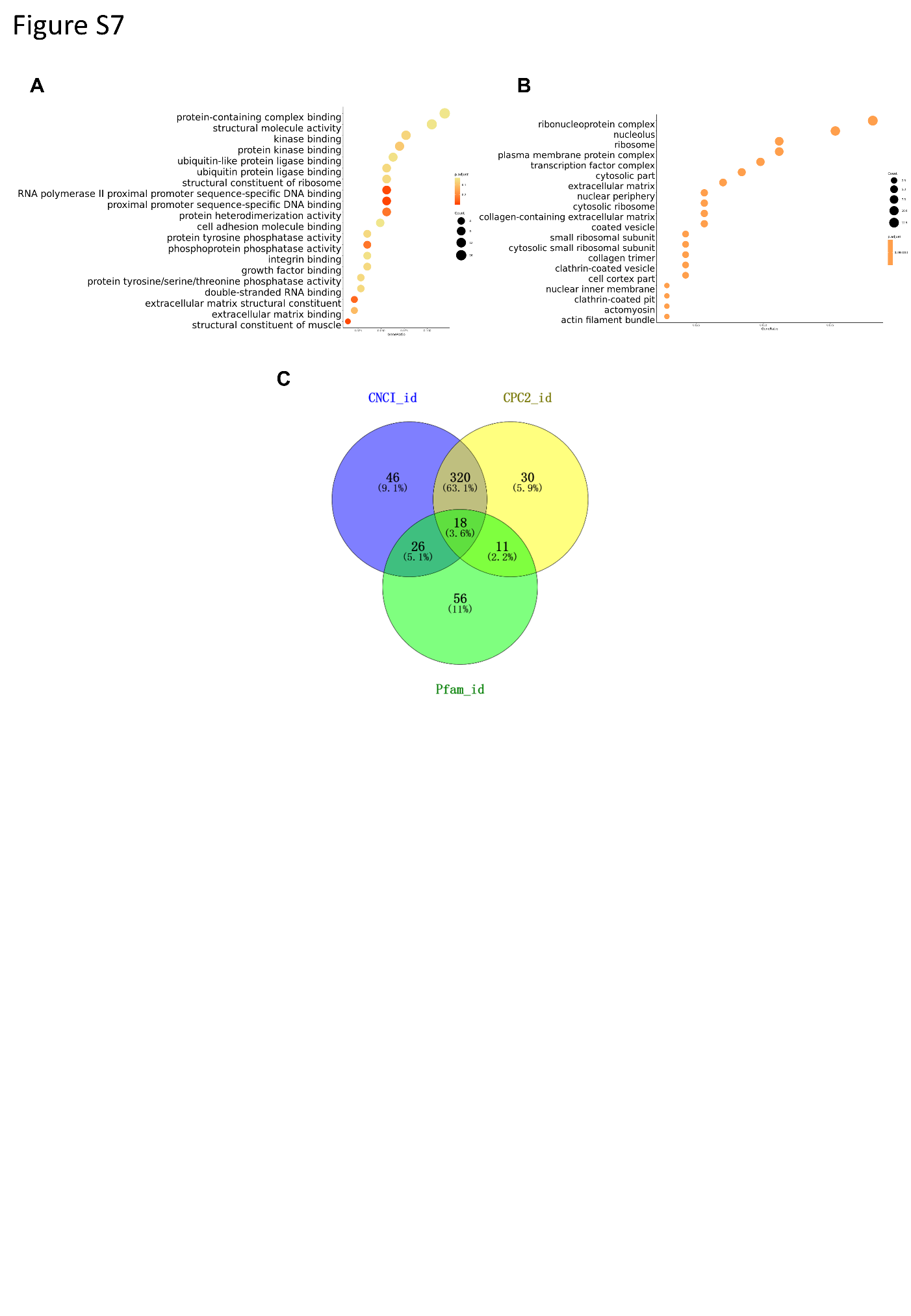
**

**Figure S7. GO term of DEmiRNA and identification of candidate lncRNA.** (A) GO Molecular Function. (B) GO Cellular Component. (C) The Venn diagram showing the selected lncRNAs.

**
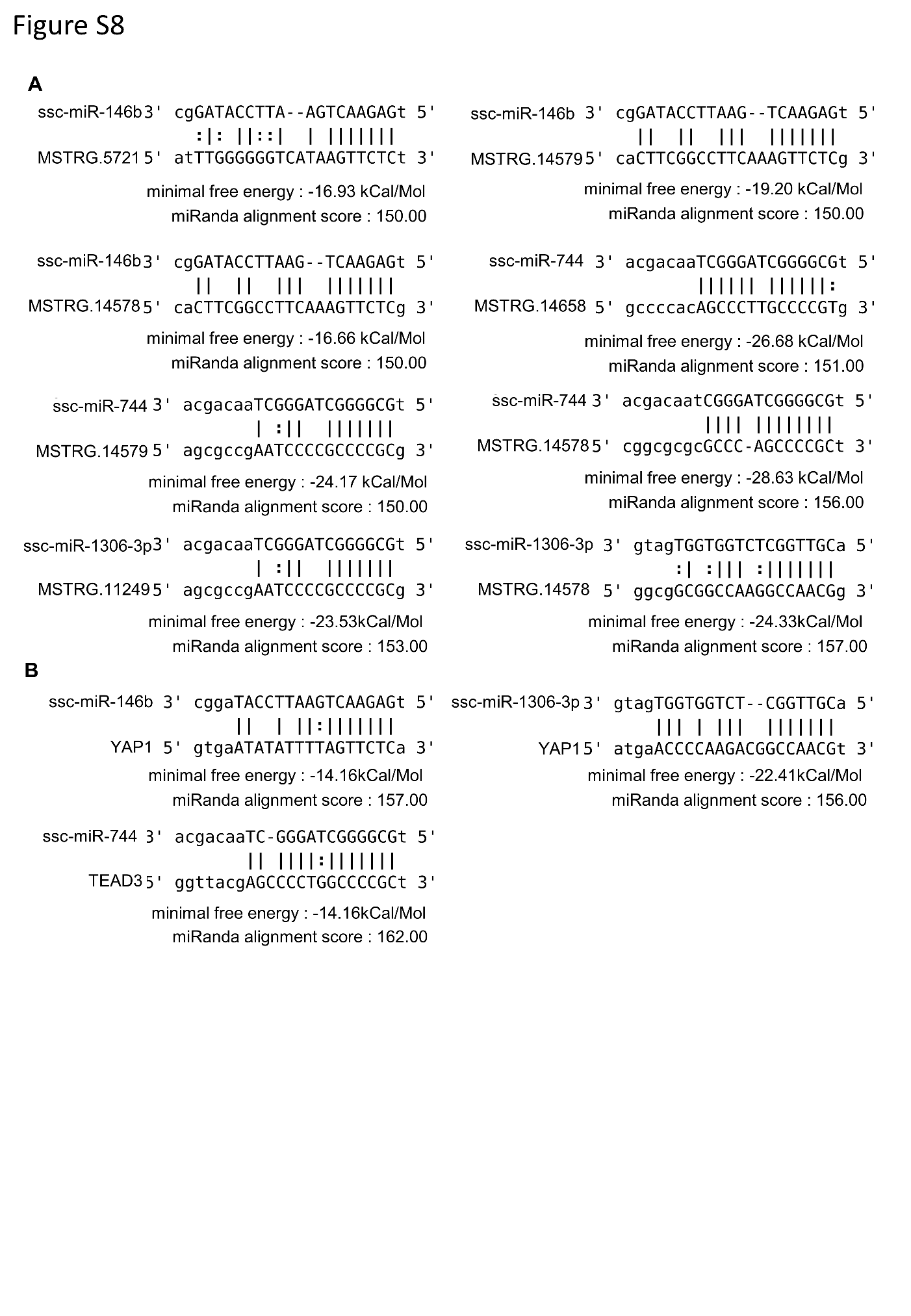
**

**Figure S8. Schematic diagram of binding sequence.** (A) Schematic diagram of the binding sequence of miRNAs and lncRNAs. (B) Schematic diagram of the binding sequence of miRNAs and mRNAs.

**Table S1.** Antibodies used in this paper

| **Primary antibodies** | | **Vendor** | **Dilution** | Source |
| --- | --- | --- | --- | --- |
| VASA(IF/Flow Cyt/WB)  PCNA (IF/Flow Cyt/WB)  TEAD3(IF/WB)  YAP1(IF/WB)  Phospho-YAP1-S127(WB)  OCT4 (IF)  SOX2 (IF)  SOX9 (IF)  SSEA-1 (IF)  NANOG (IF)  DAZL (IF)  PRDM14 (IF)  SOX17 (IF)  Caspase-3 (WB)  BCL2 (WB)  BAX (WB)  GAPDH(WB) | | Abcam (ab13840)  Abcam (ab29)  Affinity (DF4677)  Affinity (AF6328)  Affinity (AP0489)  Abcam(ab18976)  Sangon (D121248)  Affinity (AF6330)  Abcam (ab16285)  Abclonal (A3232)  Abcam (ab14139)  Abclonal (A5543)  Affinity (DF9090)  Abcam (ab49822)  Beyotime (AB112)  Cell signaling (#2772)  ImmunoWay (YM3040) | 1:200/1:200/1:200  1:200/1:200  1:200/1:1000  1:200/1:1000  1:500  1:200  1:200  1:200  1:200  1:200  1:200  1:200  1:200  1:1000  1:1000  1:1000  1:1000 | Rabbit  Mouse  Rabbit  Rabbit  Rabbit  Rabbit  Rabbit  Rabbit  Rabbit  Rabbit  Rabbit  Rabbit  Rabbit  Rabbit  Rabbit  Rabbit  Mouse |
| **Secondary antibodies**  CY5-conjugated goat anti-rabbit (IF/Flow Cyt)  FITC-conjugated goat anti-rabbit (IF/Flow Cyt)  HRP-conjugated goat anti- Mouse IgG (WB)  HRP-conjugated goat anti- rabbit IgG (WB) | Beyotime (A0516)  Beyotime (A0562)  Beyotime (A0216)  Beyotime (A0258) | | 1:200  1:200  1:1000  1:1000 | Goat  Goat  Goat  Goat |

**Table S2.** Primers Used for Quantitative RT-PCR

| Genes | Forward primer sequence | Reverse primer sequence |
| --- | --- | --- |
| GAPDH | GTCGGAGTGAACGGATTTGGC | CACCCCATTTGATGTTGGCG |
| YAP1 | ACCCTCGTTTTGCCATGAAC | TGTGCTGGGATTGATATTCCGTA |
| TEAD3 | CTGTACCCTTCCTGCGTCTG | TTGGACGCTATTGTGCTGGT |
| CCND3 | TACTTCCAATGTGTGCAGAGGG | GAGCCAGGAAATCATGGGCA |
| CDK1 | GGATCCAGGACCCTTTAGCG | TCCACTTCTGGCCACACTTC |
| CDK2 | GGTGGTTTGGCCAGGAGTTA | AGCCATAGGGAAGCACAAGC |
| P21 | TTGTATCAGGCCGCCCATTG | GCTTCGCTATGGTCCTCTCC |
| P53 | AGTCACGAACTGGCTGGATG | TTCAGCTCCAAGGCGTCATT |
| PCNA | ACACTAAGGGCCGAAGATAACG | ACAGCATCTCCAATATGGCTGA |
| OCT4 | CTATGACTTCTGCGGAGGGAT | TTTGATGTCCTGGGACTCCTCG |
| SOX2 | ATGGGCTCAGTGGTCAAGTC | AGAGAGGCAGTGTACCGTTG |
| NANOG | CTGGGACCTTTTCCTCCTTC | CATCCATTTCCAGCGAATCT |
| ssc-miR-146b | CTGAGAACTGAATTCCATAGGC |  |
| ssc-miR-744 | TTTGCGGGGCTAGGGCTAACA |  |
| ssc-miR-1306-3p | CGTTGGCTCTGGTGGTGATG |  |
| MSTRG.5721 | TAAGTCCAAACCGCTCTGCT | GGATGCTGTCACAGACGAGA |
| MSTRG.14658 | GAACTTTGAAGGCCGAAGTG | ATCTGAACCCGACTCCCTTT |
| MSTRG.14579 | CAGGGGAATCCGACTGTTTA | CGAGGCATTTGGCTACCTTA |
| MSTRG.11249 | AGCAGAGCATTCCCTTCAAA | TTATGTGACCCCTCGGAGAC |
| MSTRG.14578 | CGAGGCATTTGGCTACCTTA | CAGGGGAATCCGACTGTTTA |

**Table S3.** Genes expression in lncRNA-miRNA-mRNA network

| Genes | log2FoldChange | p.adjust |
| --- | --- | --- |
| YAP1 | 1.58317678557357 | 0.00216844122619385 |
| TEAD3 | 1.77166446090771 | 0.0015930546313161 |
| ssc-miR-146b | -1.718486024 | 0.003712367 |
| ssc-miR-744 | -1.662580682 | 0.000320754 |
| ssc-miR-1306-3p | -1.857501725 | 0.005313168 |
| MSTRG.5721 | 0.83717849 | 0.004661243 |
| MSTRG.14658 | 0.680437075 | 3.17E-05 |
| MSTRG.14579 | 0.501853399 | 8.05E-07 |
| MSTRG.11249 | 0.674094633 | 0.010436587 |
| MSTRG.14578 | 0.553428541 | 1.91E-15 |

**Table S4.** Analysis of KEGG pathways of target genes (356)

| ID | Description | p.adjust | Count |
| --- | --- | --- | --- |
| ssc04390 | Hippo signaling pathway | 0.035101061 | 15 |
| ssc04151 | PI3K-Akt signaling pathway | 0.006307907 | 11 |
| ssc04510 | Focal adhesion | 0.047588589 | 10 |
| ssc04512 | ECM-receptor interaction | 0.005510289 | 9 |
| ssc03010 | Ribosome | 0.047588589 | 8 |
| ssc04926 | Relaxin signaling pathway | 0.047156057 | 8 |
| ssc04068 | FoxO signaling pathway | 0.047156057 | 8 |
| ssc04371 | Apelin signaling pathway | 0.047588589 | 8 |
| ssc04974 | Protein digestion and absorption | 0.035101061 | 7 |
| ssc04392 | Hippo signaling pathway - multiple species | 0.035101061 | 4 |
