# Supplementary figures and images for: The proliferation role of LH on porcine primordial germ cell‐like cells (pPGCLCs) through ceRNA network construction
Source: Clin Transl Med. 2021 Oct 14;11(10):e560. doi: 10.1002/ctm2.560 (PMC8516341; doi:10.1002/ctm2.560)

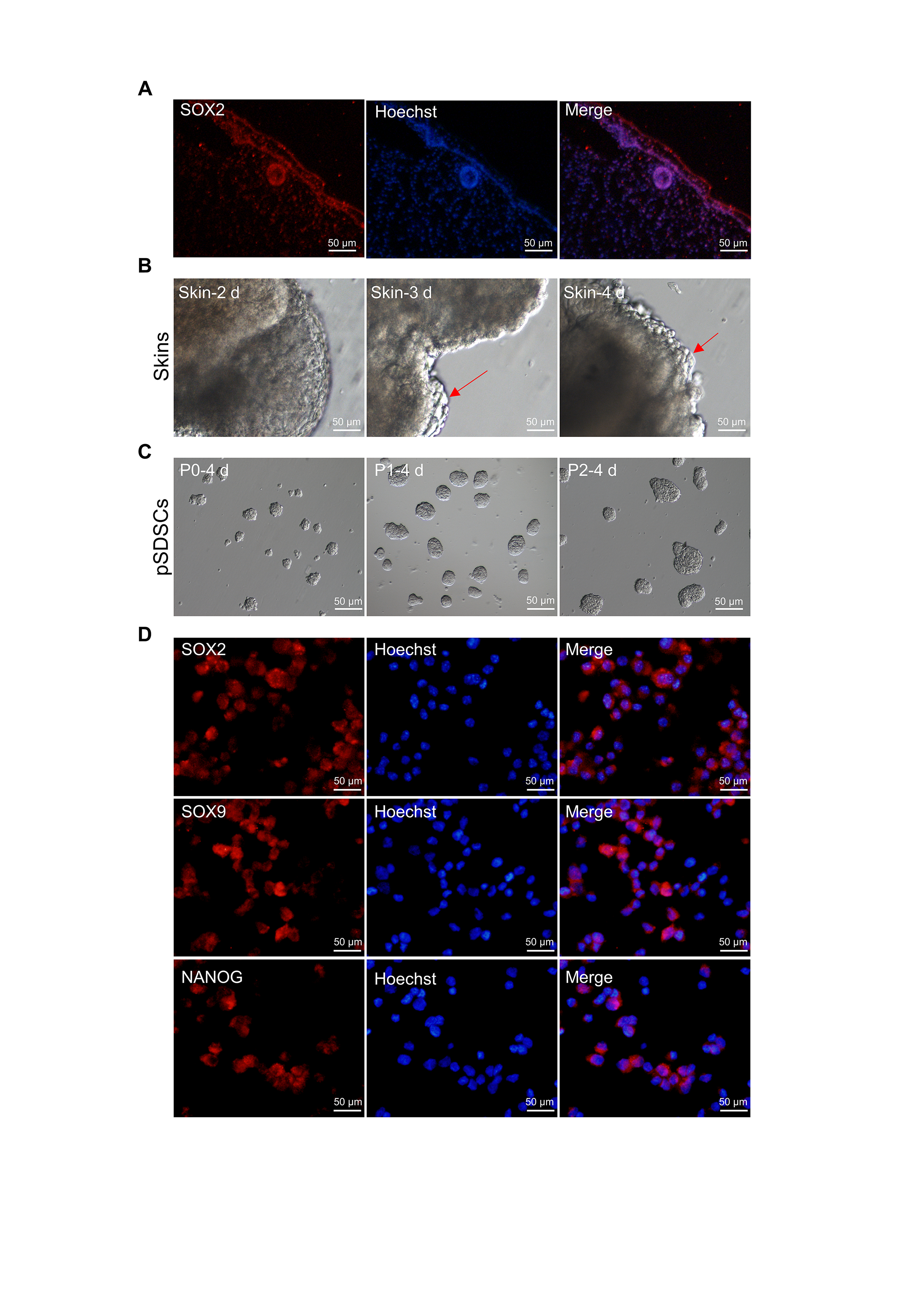

Supplement: Supplementary file 2 — Figure S1. The characterisation of pSDSCs. (A) The expression of SOX2 in the pig foetuses skin. Bar = 50 μm. (B) Morphology of pig foetuses skins at different days. Bar = 50 μm. (C) Colony morphology of pSDSCs at different passages. Bar = 50 μm. (D) SOX2, SOX9 and NANOG immunocytochemistry of pSDSCs. Bar = 50 μm [file CTM2-11-e560-s009.TIF]

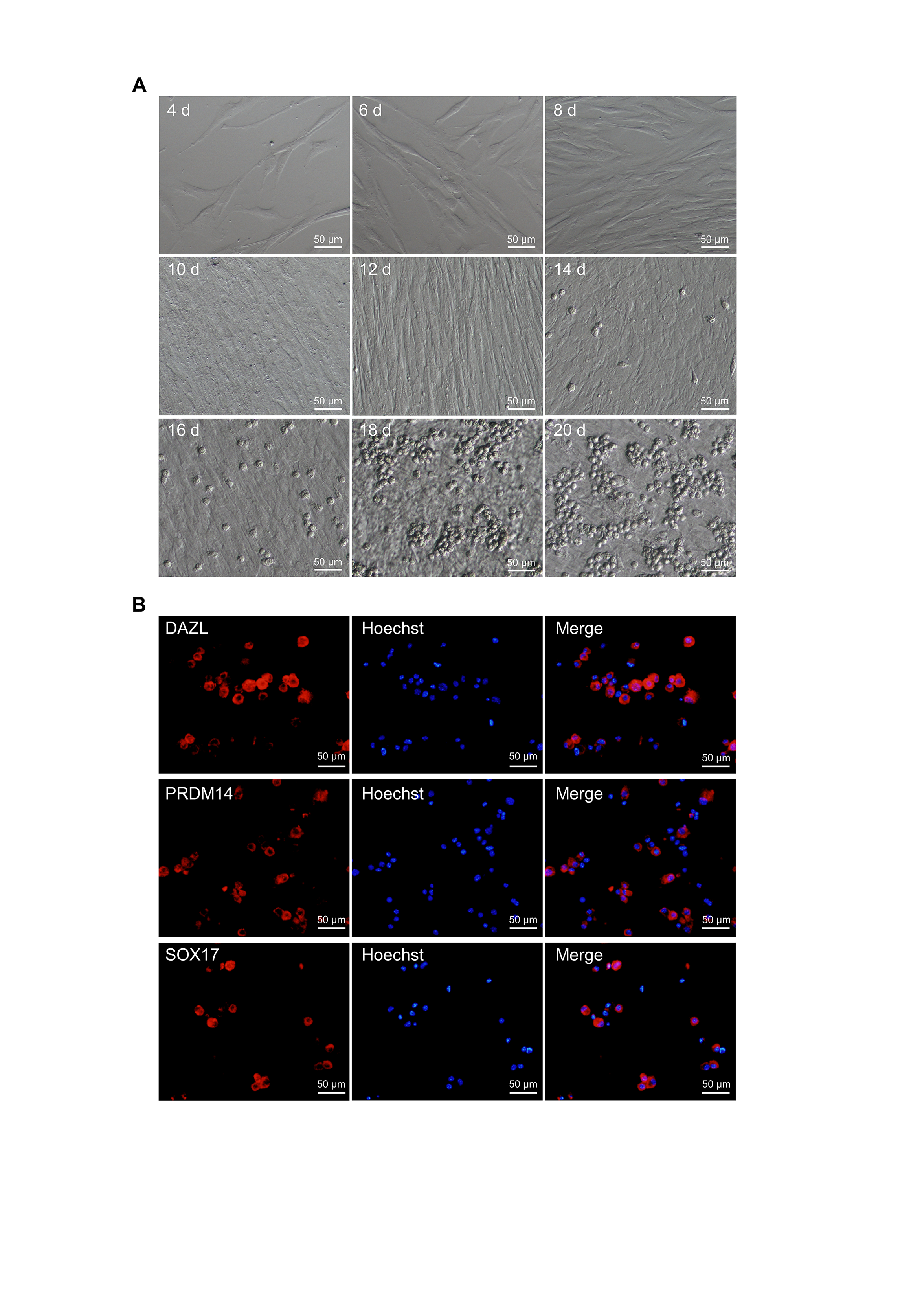

Supplement: Supplementary file 3 — Figure S2. The characterisation of pPGCLCs. (A) Different days of pPGCLCs. Bar = 50 μm. (B) DAZL, PRDM14 and SOX17 immunocytochemistry of pPGCLCs. Bar = 50 μm [file CTM2-11-e560-s004.TIF]

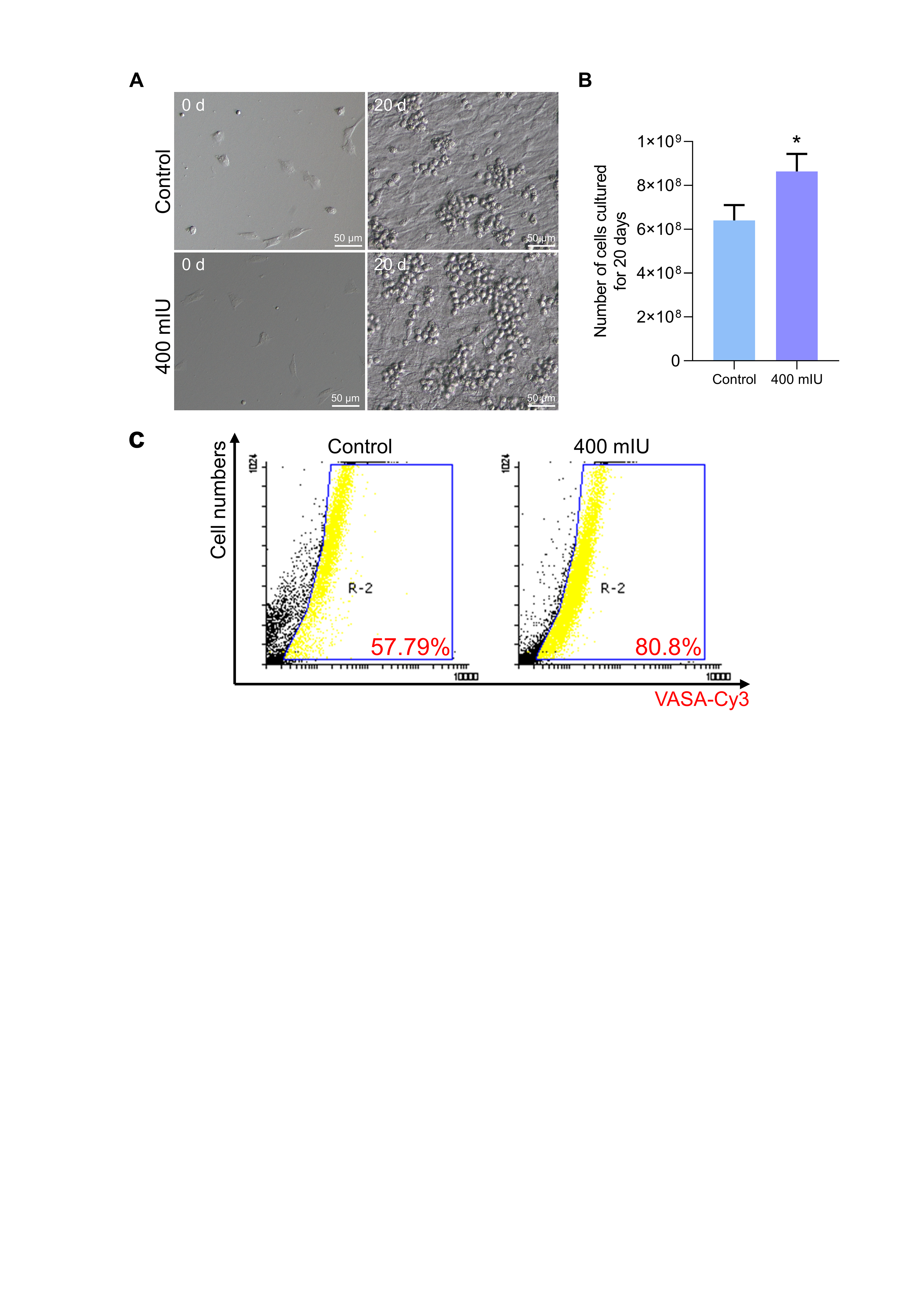

Supplement: Supplementary file 4 — Figure S3. The number of pPGCLCs increased significantly after the addition of LH. (A) Cell images of the control and LH‐treated group at 0 and 20 days. (B) Number of cells cultured for 20 days of control and LH‐treated group. (C) Flow cytometry analysis of VASA positive pPGCLCs. The results are presented as mean ± SD. *p < .05; **p < .01 [file CTM2-11-e560-s002.TIF]

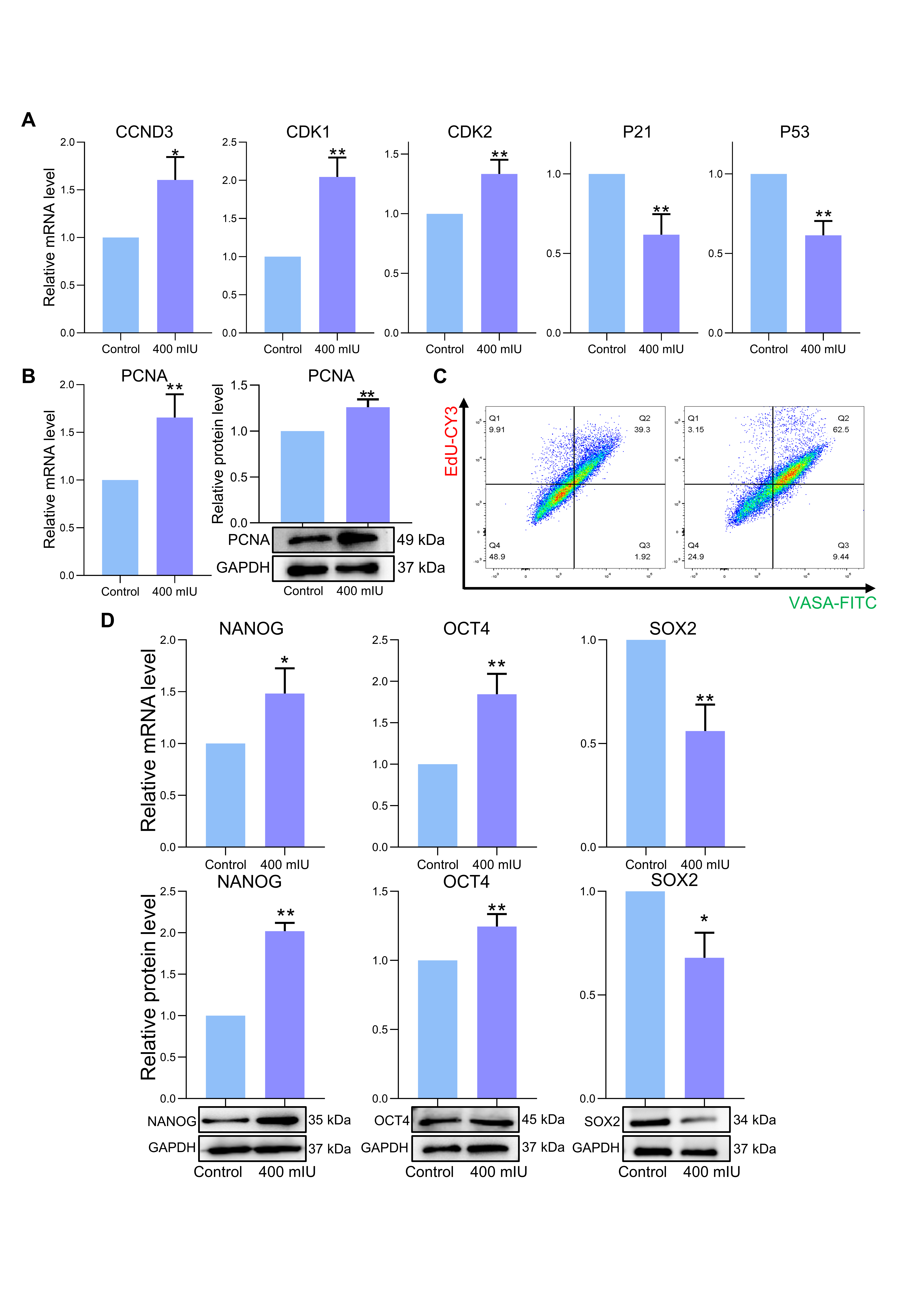

Supplement: Supplementary file 5 — Figure S4. LH promotes the proliferation of pPGCLCs. (A) Effect of LH on the levels of mRNA of proliferation genes. (B) The expression of PCNA. (C) Flow cytometry analysis of VASA and EdU double‐positive pPGCLCs. (D) Change in pluripotency of pPGCLCs. The results are presented as mean ± SD. *p < .05; **p < .01 [file CTM2-11-e560-s008.TIF]

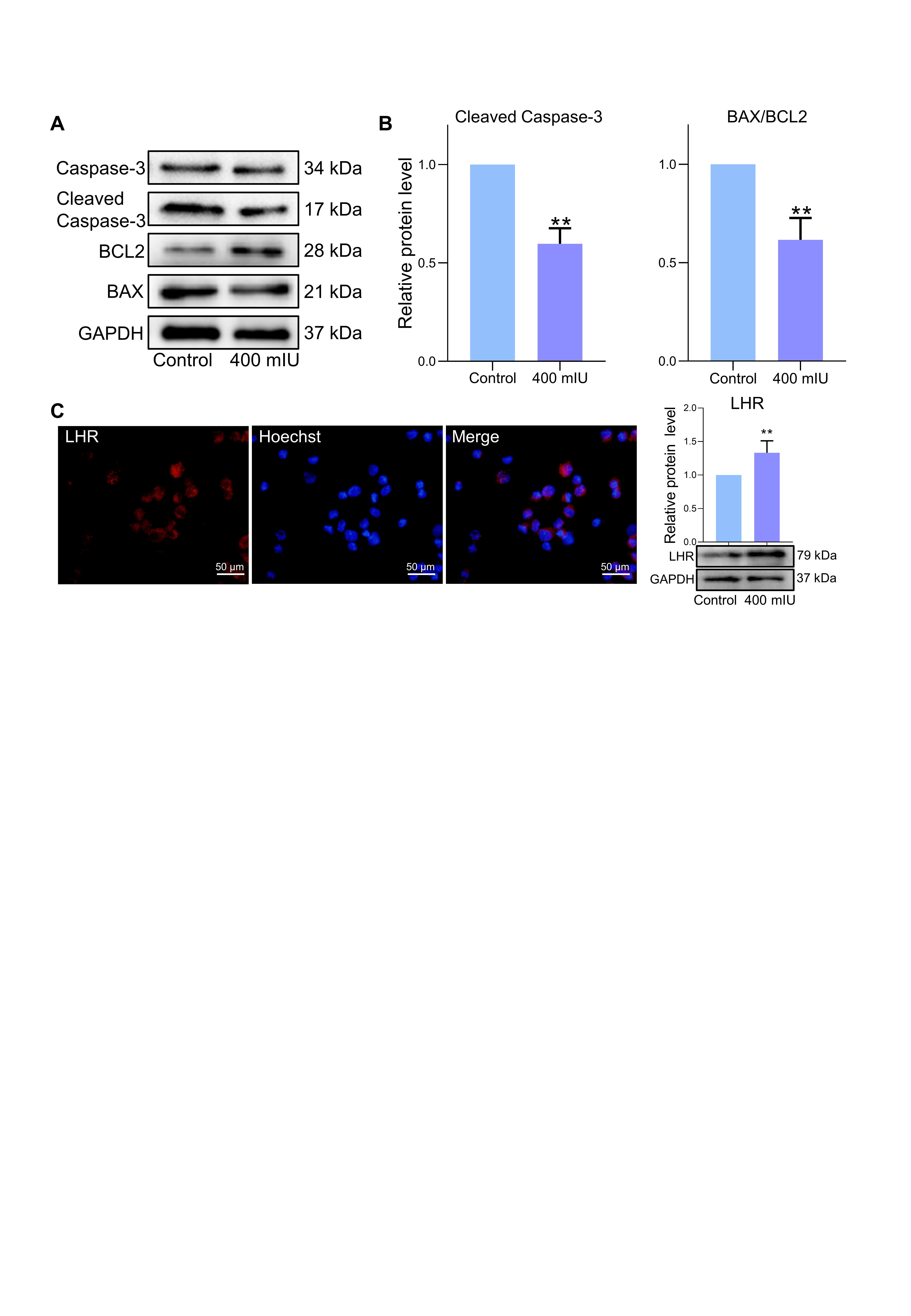

Supplement: Supplementary file 6 — Figure S5. LH‐stimulated proliferation is accompanied by decreased apoptosis. (A) WB images of BAX, BCL2, Caspase‐3 and Cleaved Caspase‐3. (B) The relative protein level of Cleaved Caspase‐3 and BAX/BCL2. (C) The expression of LHR. The results are presented as mean ± SD. *p < .05; **p < .01 [file CTM2-11-e560-s006.TIF]

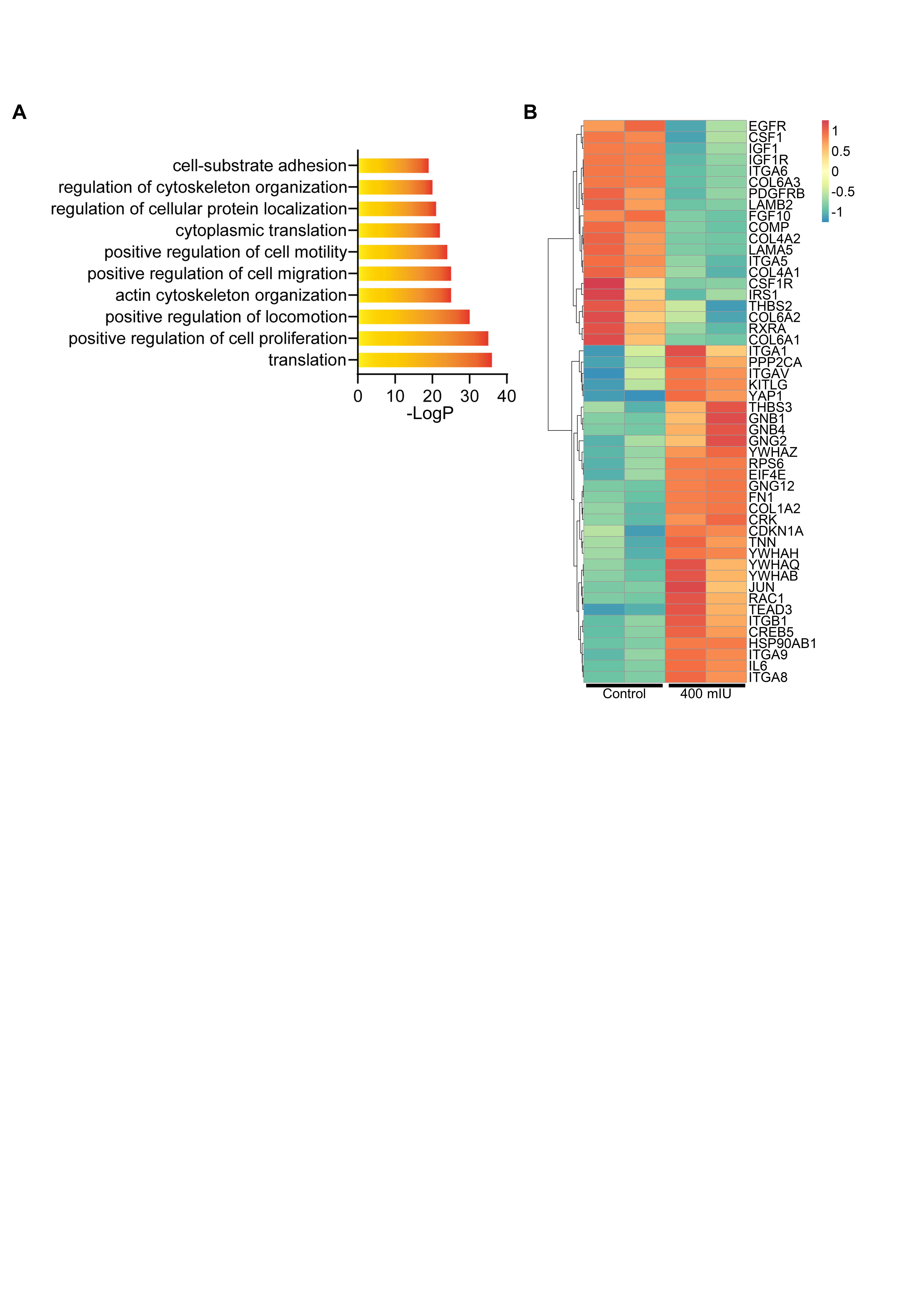

Supplement: Supplementary file 7 — Figure S6. GO enrichment results (RNA‐seq). (A) GO Biological Process. (B) Heatmap of DEmRNAs in Hippo signalling pathway [file CTM2-11-e560-s003.TIF]

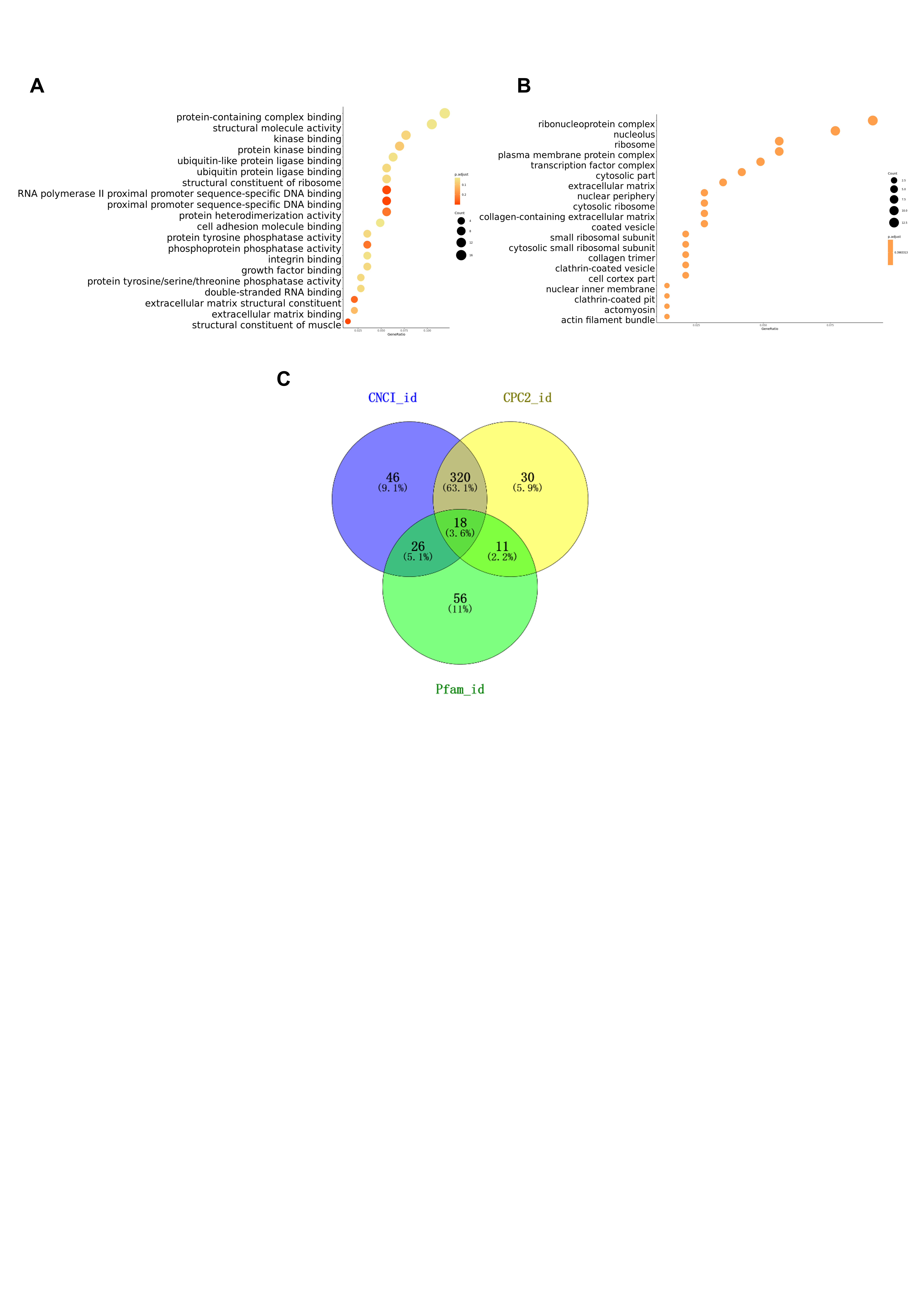

Supplement: Supplementary file 8 — Figure S7. GO term of DEmiRNA and identification of candidate lncRNA. (A) GO Molecular Function. (B) GO Cellular Component. (C) The Venn diagram showing the selected lncRNAs [file CTM2-11-e560-s007.TIF]

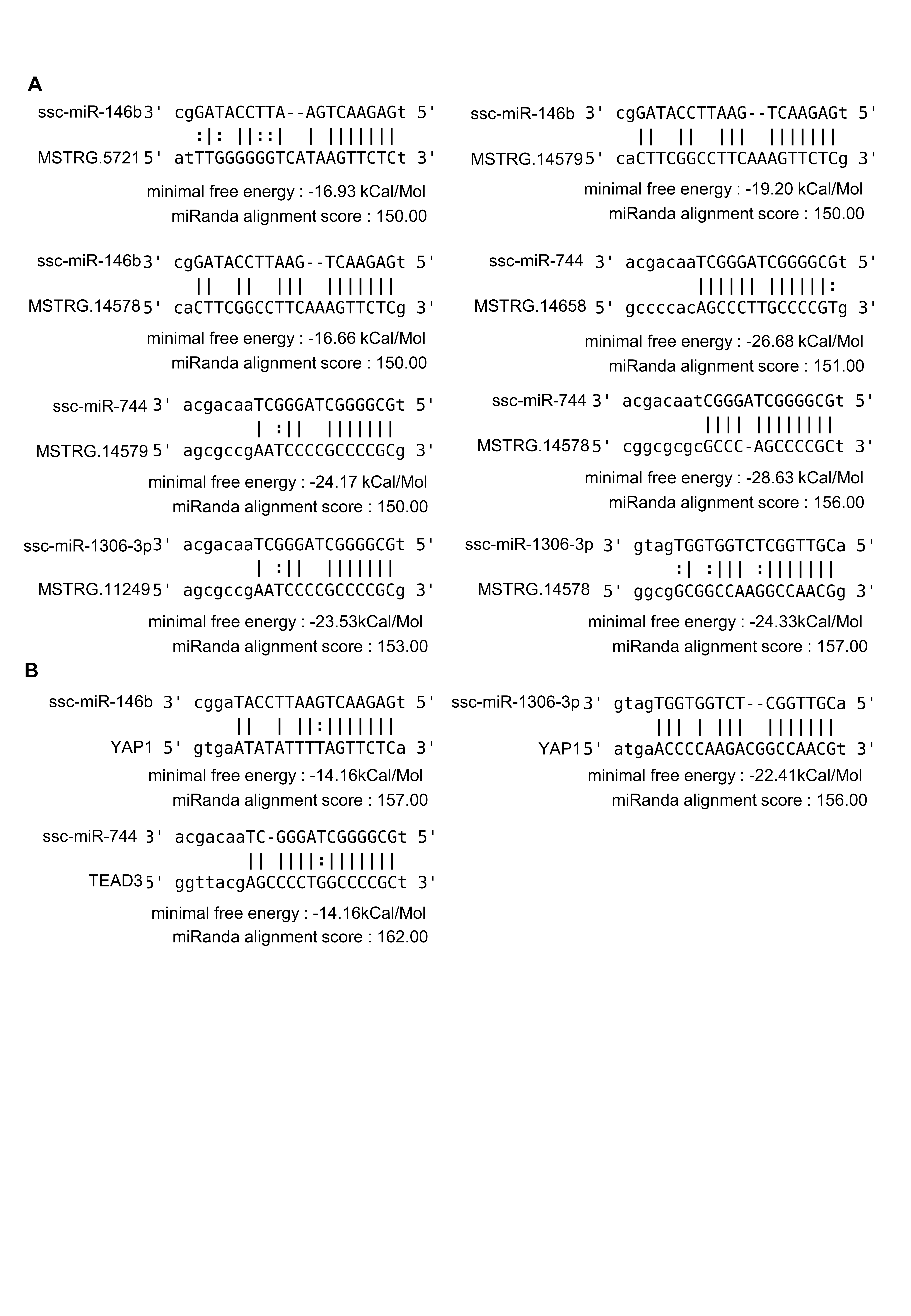

Supplement: Supplementary file 9 — Figure S8. Schematic diagram of binding sequence. (A) Schematic diagram of the binding sequence of miRNAs and lncRNAs. (B) Schematic diagram of the binding sequence of miRNAs and mRNAs [file CTM2-11-e560-s001.TIF]
